# Global prevalence of insomnia symptoms in undergraduate university students: a systematic review and meta-analysis

**DOI:** 10.1093/sleepadvances/zpaf083

**Published:** 2025-11-17

**Authors:** Spyros Spyridonidis, Dhillon Lad, Harrison Peters, Jason Ellis, Lucy J Robinson

**Affiliations:** School of Psychology, Newcastle University, Newcastle upon Tyne, United Kingdom; Cumbria, Northumberland, Tyne and Wear NHS Foundation Trust, Newcastle upon Tyne, United Kingdom; School of Psychology, Newcastle University, Newcastle upon Tyne, United Kingdom; Cumbria, Northumberland, Tyne and Wear NHS Foundation Trust, Newcastle upon Tyne, United Kingdom; School of Psychology, Newcastle University, Newcastle upon Tyne, United Kingdom; Cumbria, Northumberland, Tyne and Wear NHS Foundation Trust, Newcastle upon Tyne, United Kingdom; Department of Psychology, Northumbria University, Newcastle upon Tyne, United Kingdom; School of Psychology, Newcastle University, Newcastle upon Tyne, United Kingdom

**Keywords:** sleep initiation and maintenance disorders, prevalence, epidemiology, meta-analysis

## Abstract

Insomnia is increasingly recognized as a public health concern; however, undergraduate university students remain relatively understudied. This study aimed to estimate the overall pooled prevalence of insomnia symptoms in this population to inform the need for targeted care. For this systematic review and meta-analysis, we searched five databases to identify papers published between January 1, 1993, and January 17, 2025, which investigated the prevalence or proportion of insomnia in undergraduate university students using validated measures. Studies with biased samples were excluded. Study quality was assessed using the Joanna Briggs Institute Critical Appraisal Tool. Data were synthesized using random-effects meta-analysis and meta-regression. Subgroup analyses were conducted based on continent, screening instrument, field of study, and sampling method. The review was registered on PROSPERO (CRD42025617914). The search yielded 2379 non-duplicate citations, of which 48 met the inclusion criteria, contributing 55 prevalence estimates based on data from 95 938 students. The pooled prevalence of insomnia in undergraduate university students was 46.9% (95% CI = 40.1% to 53.6%). Heterogeneity was high (*I*^2^ = 99.8%). Meta-regression indicated that rates varied by continent and screening instrument. Findings suggest that nearly half of undergraduate students experience insomnia symptoms, highlighting the need for university-level responses that combine universal sleep health promotion with targeted interventions. Further methodologically rigorous and culturally sensitive research is required to guide policy and practice.

## Introduction

Insomnia is a major public health issue [1]. According to the Diagnostic and Statistical Manual of Mental Disorders (DSM-5), insomnia disorder is defined as a persistent dissatisfaction with the quality or quantity of sleep, characterized by difficulties in initiating or maintaining sleep, or by early-morning awakenings that result in significant distress and daytime impairment. These symptoms must occur at least three nights per week and persist for a minimum of 3 months [2]. Approximately one-third of the general population reports experiencing insomnia symptoms, with an estimated 6% to 10% meeting the diagnostic criteria for chronic insomnia [1]. However, prevalence rates vary widely across countries and studies, largely due to differences in sampling methods, case definitions, diagnostic criteria, and standardized assessment procedures [1, 3, 4]. Women [5], older adults [6], and individuals from lower socioeconomic backgrounds [7] appear to be at increased risk, as do those with coexisting physical and mental health conditions [8].

Although there is growing interest in the epidemiology of insomnia in both general and clinical populations [1, 6], university students represent a distinct subgroup that has received comparatively limited attention. This is despite clear evidence that sleep problems are common at university entry and persist throughout the academic year [9]. The transition to university occurs during a critical developmental period [10] and introduces a number of unique factors that may precipitate poor sleep quality and insomnia, including new living arrangements, environmental noise, social pressure, unstructured academic lifestyle, academic stress, limited social support, increased independence, substance use, and irregular sleep–wake patterns [11, 12]. In university students, insomnia is associated with numerous adverse outcomes, including anxiety and depression [13], stress [14], increased suicide risk [15], and lower levels of academic achievement [16, 17]. More broadly, insomnia is linked to a range of somatic and mental disorders [18, 19], fatigue [20], reduced quality of life [21], and significant economic impact [22].

Understanding the epidemiological landscape of insomnia among undergraduate students is important for evaluating the demand for care and guiding the development and dissemination of scalable evidence-based sleep interventions and prevention strategies within university support services. The higher education setting presents a critical window of opportunity to identify and address sleep difficulties, thereby mitigating the adverse effects of insomnia on academic performance and overall health [23]. While several reviews have investigated the prevalence of insomnia among university student populations in Saudi Arabia [24], South Asia [25], and worldwide [26], they have limitations in terms of the assessment of insomnia using measures that lack specificity for insomnia. Although they suggest estimates of insomnia are high, ranging from 11.2% to 63.1%, and vary considerably across demographic and geographical contexts, a systematic synthesis of the evidence base specifically focusing on undergraduate students across the globe has yet to be conducted.

The aim of the present study was to synthesize the accumulating research on the global prevalence of insomnia in undergraduate university students and investigate whether study-level characteristics including year of publication, continent, screening instrument, field of study, sampling method, proportion of female participants, or risk of bias score accounted for any observed variation.

## Materials and Methods

A systematic literature search was conducted following the Preferred Reporting Items for Systematic Reviews and Meta-Analyses (PRISMA) statement (Table S1) [27]. The protocol for this review was prospectively registered with PROSPERO (CRD42025617914) before data extraction.

### Search strategy

Five electronic databases: MEDLINE, Embase, Web of Science, PsycINFO, and CINAHL were used. The review was restricted to studies published between January 1, 1993 and January 17, 2025. To minimize the possibility of missing relevant data, we hand-searched reviews and meta-analyses pertinent to our objectives, as well as the bibliographies of included studies, and directly contacted authors to request data, where appropriate. The strategy was adapted for each database and included terms for (i) insomnia disorder, (ii) prevalence, and (iii) university students (see Tables S2–S6 for more details on search strategy).

### Eligibility criteria

Studies were considered eligible if they: (a) were first-contact and contained original cross-sectional data (for longitudinal studies, only baseline data were considered); (b) reported prevalence estimates or provided sufficient data to derive prevalence (e.g. numerator and denominator); (c) sampled undergraduate university students, including medical and nursing, or a mixed sample (e.g. undergraduates and postgraduates) with findings reported separately for undergraduates; (d) used standardized instruments with cutoffs that map onto diagnostic manual categories, such as the International Classification of Diseases (ICD-10, ICD-11), Diagnostic and Statistical Manual of Mental Disorders (DSM-IV, DSM-5), or the International Classification of Sleep Disorders (ICSD-2, ICSD-3); (e) were published from January 1, 1993 (publication of the ICD-10) to January 17, 2025; and (f) were published online in the scientific literature. Studies published in languages other than English were included, with translations conducted using Google Translate.

We excluded (a) qualitative studies, study protocols, book chapters, editorial letters, review articles, and conference abstracts; (b) studies utilizing non-standardized measures or indirect methods to assess insomnia (e.g. sleep quality or sleep disturbance measures); (c) studies using potentially biased samples (e.g. only female students); and (d) studies sampling both undergraduate and postgraduate students or vocational college students, where data for undergraduate students could not be extrapolated.

### Study selection

The resulting list of articles was deduplicated using Endnote 21 [28] and all references were imported into Rayyan [29] for title, abstract, and full-text screening. Three authors (S.S., D.L., H.P.) screened the titles found to assess whether they met eligibility criteria using a predetermined form covering inclusion criteria and guidance notes (inter-rater agreement = 97.94% and Cohen’s *κ* = .93). Reviewers remained blind to each other’s decisions until all records had been reviewed in duplicate. Studies were rated as either “did not meet initial eligibility criteria,” “met initial eligibility criteria,” or “possibly met initial eligibility criteria.” We excluded citations which did not meet inclusion by consensus and the remaining studies were forwarded to duplicate independent abstract review, and if appropriate, full-text review (inter-rater agreement = 95.1% and Cohen’s *κ* = .77). Disagreements about inclusion were resolved through discussion and consensus. All retrieved articles were stored in Microsoft Excel for data extraction, while excluded studies were coded to indicate the primary reason for exclusion (see Table S7).

### Data extraction

Two pairs of investigators (S.S., H.P., D.L., N.W.) independently extracted the following data from each article using a standardized form: study title, first author, year of publication, location, study period, design, population characteristics, sample size, mean age and age range, gender distribution, insomnia prevalence along with the corresponding 95% confidence interval (CI), screening instrument including cutoffs used, response rate, and other relevant information. Uncertainties were resolved through discussion and consensus. For studies with poorly reported outcomes or where different cutoffs were used, the corresponding authors were contacted for clarification or to request additional data (*n* = 26). Seventeen authors responded to our correspondence. When multiple studies were based on the same sample, we included only the most comprehensive study to avoid non-independent samples.

### Risk of bias assessment

One researcher (S.S.) independently assessed the risk of bias using the Joanna Briggs Institute’s Critical Appraisal Checklist for Studies Reporting Prevalence Data [30]. Two researchers (D.L., H.P.) screened a randomly selected 50% sample and any disagreements were resolved through discussion and consensus. Quality assessment criteria include (a) representativeness of the target population, (b) recruitment of participants, (c) adequacy of sample size, (d) description of study participants and setting, (e) coverage of the identified sample, (f) objectivity of assessment, (g) reliability of the assessment, (h) appropriateness of statistical analysis, and (i) response rate. This instrument consists of nine questions answered as “yes,” “no,” “unclear,” or “not applicable,” with a higher number of “yes” responses indicating a lower risk of bias. Quality assessment findings were summarized by tabulating the results (Table S8). Publication bias testing was not undertaken as it is not recommended for meta-analyses of proportional or prevalence estimates [31].

### Data synthesis

We first conducted a narrative synthesis to summarize the included studies. Due to the anticipated high levels of heterogeneity because of the multiple sources of variance, we used a random-effects meta-analysis a priori to quantify this heterogeneity. We transformed prevalence estimates using the Freeman−Tukey double arcsine transformation to stabilize variance and to prevent CIs from extending beyond 0% and 100%. After analyses, we back-transformed the results to prevalence rates with 95% CIs for ease of interpretation [32]. The data were displayed graphically in forest plots of prevalence rates and 95% CΙs [33].

We assessed statistical heterogeneity using the Cochran’s *Q* test and quantified it with the *I*^2^ statistic [34]. An *I*^2^ value ≥75% was considered to represent considerable heterogeneity [35], although this is expected in prevalence meta-analyses [36]. We used stratified meta-analyses to compare studies according to prespecified study-level characteristics (continent, screening instrument, field of study, sampling method) and random-effects meta-regression to explore whether heterogeneity was associated with the year of publication, continent, screening instrument, field of study, sampling method, proportion of female participants, or risk of bias score. Lastly, we performed a leave-one-out sensitivity analysis to assess the influence of individual studies on overall pooled estimates by serially excluding studies one by one. We conducted meta-analyses in Stata, version 18, using the “metaprop” command and meta-regressions using the “meta regress” command.

## Results

### Study selection

**Figure 1:**
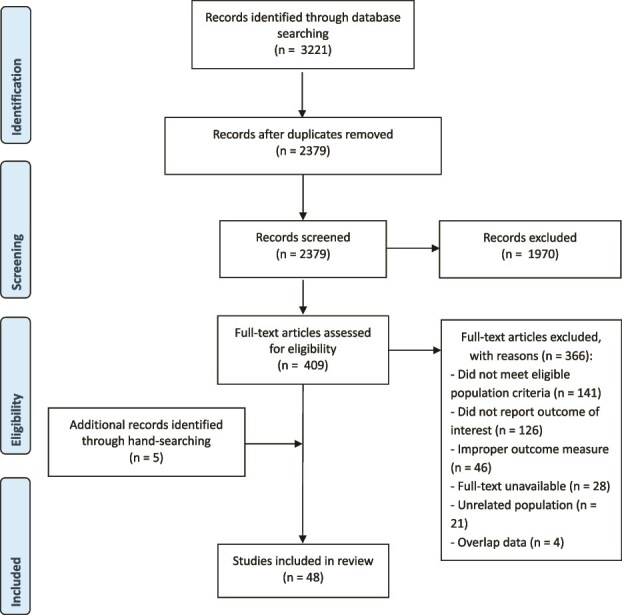
PRISMA flow diagram.

We retrieved 3221 records in the five electronic databases. After removing duplicates, there were 2379 publications for title/abstract screening and 409 studies were identified for a detailed, full-text assessment. Hand searching of the reference lists and citations of the included studies revealed five additional studies that met inclusion criteria. In total, 48 studies fulfilled the inclusion criteria, with 55 separate estimates (Figure 1 and Table 1).

**Table 1:** Characteristics of the Studies Included in the Meta-Analysis

No.	**Study (first author, pub. date)**	**Location**	**Period**	**Sample size (*n*)**	**Sampling method**	**Field of study**	**Mean age (*SD*)**	**% Females**	**Insomnia assessment**
1	Akram et al. (2023) [64]	UK	2018–2019	1213	Non-probability	Mixed sample	20.17 (3.56)	82.70%	SCI ≤ 16
2	Al Maqbali et al. (2023) [65]^a^	Saudi Arabia	2022	442	Non-probability	Nursing	NR	85.30%	ISI ≥ 10
	Al Maqbali et al. (2023) [65]^b^	Oman	2022	289	Non-probability	Nursing	NR	78.90%	ISI ≥ 10
	Al Maqbali et al. (2023) [65]^c^	UK	2022	97	Non-probability	Nursing	NR	95.90%	ISI ≥ 10
	Al Maqbali et al. (2023) [65]^d^	UAE	2022	90	Non-probability	Nursing	NR	98.90%	ISI ≥ 10
3	Al Omari et al. (2022) [66]^*^	Oman	2021	632	Non-probability	Mixed sample	21.53 (1.68)	41.10%	ISI ≥ 10
4	Alkhatatbeh et al. (2021) [67]^*^	Jordan	2019–2020	1000	Non-probability	Mixed sample	20·87 (2.69)	63.30%	ISI ≥ 10
5	Alqudah et al. (2019) [68]^*^	Jordan	2018	977	Non-probability	Medical and paramedical	20.90 (2.20)	63.10%	ISI ≥ 10
6	Alrashed et al. (2021) [69]	Saudi Arabia	2020–2021	463	Non-probability	Medical	NR	44.70%	ISI ≥ 8
7	Alrashed et al. (2022) [70]	Saudi Arabia	2021–2022	361	Non-probability	Medical	NR	59.60%	ISI ≥ 8
8	Alsamhori et al. (2024) [71]^*^	Jordan	2023–2024	1181	Non-probability	Mixed sample	20.43 (1.88)	74.20%	ISI ≥ 10
9	Alshammari et al. (2023) [72]	Saudi Arabia	2022	495	Non-probability	Mixed sample	20.89 (2.01)	61.84%	AIS ≥ 6
10	Alshehri et al. (2024) [73]	Saudi Arabia	2022	397	Non-probability	Medical	NR	76.60%	AIS ≥ 6
11	Arbués et al. (2019) [74]^*^	Spain	2018	1055	Non-probability	Mixed sample	21.74 (3.15)	70.50%	ISI ≥ 10
12	Babicki et al. (2023) [75]	60 countries	2016–2021	20 139	Non-probability	Mixed sample	22.60 (3.60)	78.20%	AIS ≥ 6
13	Benham (2021) [76]^*^	USA	NR	507	Non-probability	Mixed sample	22.92 (5.11)	79.00%	ISI ≥ 10
14	Bodys-Cupak et al. (2022) [77]	Poland	2020	397	Non-probability	Nursing	NR	93.70%	AIS ≥ 6
15	Chakraborty (2022) [78]	India	2019	100	Probability	Medical	22.31 (2.80)	41.00%	AIS ≥ 6
16	Chen et al. (2023) [79]	China	2023	15 803	Non-probability	Mixed sample	NR	NR	ISI ≥ 8
17	Dabrowska-Galas et al. (2021) [80]	Poland	2019	308	Non-probability	Medical	20.00 (1.20)	56.80%	AIS ≥ 6
18	Dongol et al. (2022) [81]^*^	Egypt	2020	2474	Non-probability	Mixed sample	20.40 (1.50)	68.20%	ISI ≥ 10
19	Doss et al. (2022) [82]	India	2020	400	Probability	Mixed sample	NR	59.00%	ISI ≥ 8
20	Duran-Aguero et al. (2019) [83]^*^	Chile	2017–2018	1275	Non-probability	Mixed sample	21.70 (2.40)	74.00%	ISI ≥ 10
21	Eleftheriou et al. (2021) [84]	Greece	2021	559	Non-probability	Medical	NR	69.50%	AIS ≥ 6
22	Gaultney (2010) [85]	USA	2007–2008	1845	Non-probability	Mixed sample	20.38 (4.63)	71.00%	SLEEP-50 ≥ 19
23	Goweda et al. (2020) [86]	Saudi Arabia	2020	438	Non-probability	Medical	NR	50.50%	SLEEP-50 ≥ 19
24	Gress-Smith et al. (2015) [87]^*^	USA	2009–2010	1338	Non-probability	Mixed sample	18.70 (1.00)	55.00%	ISI ≥ 10
25	Gruba et al. (2021) [88]	Poland	2021	1323	Non-probability	Mixed sample	22.23 (NR)	77.17%	AIS ≥ 6
26	Hussein et al. (2024) [89]^*^	Saudi Arabia	NR	157	Non-probability	Medical	23.52 (2.61)	78.30%	ISI ≥ 10
27	Iqbal et al. (2023) [90]	Malaysia	2021	472	Probability	Mixed sample	NR	81.60%	ISI ≥ 8
28	Islam (2021) [91]	South Korea	NR	188	Probability	Mixed sample	NR	49.50%	AIS ≥ 6
29	Ito et al. (2022) [92]^*^	Japan	2021	1197	Non-probability	Nursing	NR	94.10%	ISI ≥ 10
30	Kandeger et al. (2018) [93]^*^	India	NR	383	Non-probability	Mixed sample	21.10 (0.10)	60.10%	ISI ≥ 10
31	Kim et al. (2022) [94]^a^^*^	USA	2019	3643	Non-probability	Mixed sample	NR	73.11%	ISI ≥ 10
	Kim et al. (2022) [94]^b^^*^	USA	2020	4970	Non-probability	Mixed sample	NR	69.65%	ISI ≥ 10
32	King et al. (2023) [9]^a^	Canada	2018	2501	Non-probability	Mixed sample	18.20 (2.00)	68.00%	SCI ≤ 16
	King et al. (2023) [9]^b^	Canada	2019	2575	Non-probability	Mixed sample	18.00 (1.10)	68.10%	SCI ≤ 16
	King et al. (2023) [9]^c^	Canada	2020	1201	Non-probability	Mixed sample	18.70 (2.50)	67.30%	SCI ≤ 16
	King et al. (2023) [9]^d^	Canada	2021	1500	Non-probability	Mixed sample	18.70 (2.10)	72.80%	SCI ≤ 16
33	Liao et al. (2022) [95]^*^	China	2020	863	Non-probability	Medical	20.62 (1.45)	61.65%	ISI ≥ 10
34	Liu et al. (2022) [96]	China	2020	2741	Non-probability	Medical	NR	65.20%	AIS ≥ 6
35	Meneo et al. (2024) [97]^*^	Italy	2022	548	Non-probability	Mixed sample	23.48 (2.77)	80.50%	ISI ≥ 10
36	Morales-Suarez-Varela et al. (2024) [98]	Spain	2011–2022	1380	Non-probability	Mixed sample	NR	75.80%	AIS ≥ 6
37	Nadorff et al. (2011) [99]^*^	USA	2008	581	Non-probability	Mixed sample	19.40 (1.70)	77.35%	ISI ≥ 10
38	Parmar and Kumbhakar (2022) [100]	India	2021	385	Non-probability	Nursing	NR	54.50%	ISI ≥ 8
39	Piro et al. (2018) [101]	Iraq	2016–2017	316	Probability	Medical	20.35 (1.92)	57.00%	SLEEP-50 ≥ 19
40	Ramon-Arbues et al. (2020) [102]	Spain	2018–2019	1074	Non-probability	Mixed sample	21.73 (5.12)	71.00%	ISI ≥ 8
41	Shakeel et al. (2019) [103]	Pakistan	2019	135	Probability	Medical	21.69 (1.32)	44.44%	AIS ≥ 6
42	Solanki et al. (2023) [104]	India	2021	400	Probability	Medical	20.00 (1.58)	48.30%	ISI ≥ 8
43	Sy et al. (2024) [105]^*^	USA	2021–2022	1001	Non-probability	Mixed sample	19.9 (2.30)	71.00%	ISI ≥ 10
44	Tanji and Kodama (2021) [106]	Japan	2020	289	Non-probability	Nursing	NR	91.70%	AIS ≥ 6
45	Toubasi et al. (2023) [107]	Jordan	NR	509	Non-probability	Medical	20.66 (1.67)	49.70%	ISI ≥ 8
46	Vilca et al. (2023) [108]	Peru	NR	947	Non-probability	Mixed sample	21.60 (3.40)	58.40%	ISI ≥ 10
47	Xu and Jiang (2023) [109]	China	2019	8095	Non-probability	Mixed sample	NR	55.60%	AIS ≥ 6
48	Zhang et al. (2023) [110]	China	2022	2289	Probability	Medical	20.52 (1.93)	66.00%	AIS ≥ 6

*N*: number of cases; NR: not reported; *SD*: standard deviation. Superscript letters (a, b, c, d) indicate distinct cohorts or samples within the same study.

^^*^^Denotes studies for which supplementary data were provided by the authors.

### Study characteristics

Citations were published between 2010 and 2024 and contained data on insomnia collected between 2007 and 2024. Most of the studies were carried out in Asia (*n* = 27; 56.3%), Europe (*n* = 9; 18.8%), and North America (*n* = 7; 14.6%), while only two studies originated from South America and one from Africa. Two citations covered more than one continent, and no studies were identified from Australia. Sample sizes ranged from 100 to 20 139, with a total sample of 95 938 undergraduate university students. Thirty three of the 55 samples reported an estimate for mean sample age, which ranged from 18.0 to 23.5 years. All studies included both male and female participants, with the proportion of females ranging from 41.0% to 94.1%. The majority of studies had samples with a higher representation of female participants. Most studies included mixed samples of undergraduate university students (*n* = 28; 58.3%), while 15 (31.3%) studies focused on medical students, and 5 (10.4%) on nursing students. All studies used self-reported questionnaires, and most employed non-random sampling methods (*n* = 40; 83.3%). Twenty-eight studies (58.4%) used the Insomnia Severity Index (ISI) [37]; as a screening tool for insomnia; of these, 19 (39.6%) used a cutoff of ISI ≥ 10, and 9 (18.8%) used ISI ≥ 8. The remaining studies used the Athens Insomnia Scale (AIS) [38]; (*n* = 15; 31.3%), the SLEEP-50 [39] (*n* = 3; 6.1%), or the Sleep Condition Indicator (SCI) [40]; (*n* = 2; 4.2%). Study quality ranged from five to nine out of nine, with a median of six (interquartile range [IQR]: 5–7).

### Prevalence of insomnia in undergraduate university students

**Figure 2:**
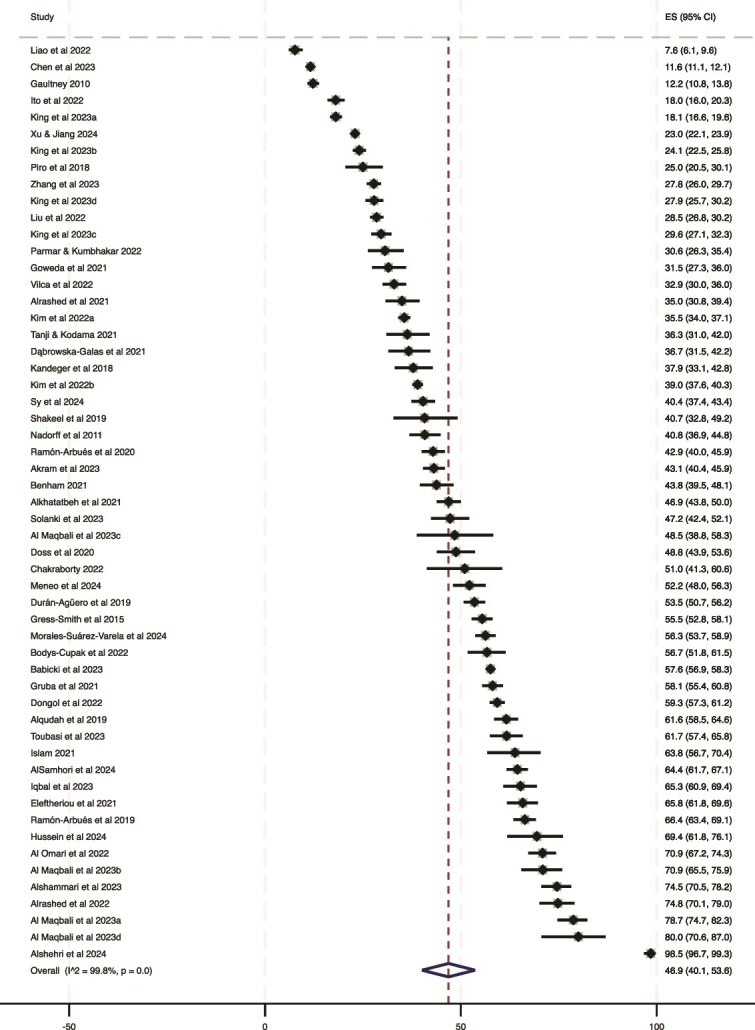
Prevalence estimates.

We included 55 separate estimates of the prevalence of insomnia derived from 48 citations (Figure 2). Prevalence rates varied almost 13 times, ranging from 7.6% (95% CI = 6.1% to 9.6%) in a sample of undergraduate medical students in China to 98.5% (95% CI = 96.7% to 99.3%) in a sample of medical students in Saudi Arabia. The overall pooled prevalence of insomnia in undergraduate university students was 46.9% (95% CI = 40.1% to 53.6%). There was evidence of heterogeneity between rates (*I*^2^  =  .99), which is unsurprising given the complex and variable epidemiology of insomnia [1], and for which we considered possible explanations (e.g. year of publication, continent, screening instrument, field of study, sampling method, proportion of female participants, or risk of bias; see below).

### Subgroup analyses

#### Continent

Europe had the highest estimated prevalence at 52.8% (95% CI = 46.7% to 58.9%), while North America had the lowest at 33.3% (95% CI = 25.9% to 40.6%). Prevalence in Asia was 49.4% (95% CI = 38.1% to 60.7%), with similar levels observed in South America at 44.1% (95% CI = 42.1% to 46.2%), although this was based on only two studies (see Table 2). A single study conducted in Africa reported a prevalence of 59.3% (95% CI = 57.3% to 61.2%), and one study spanning multiple continents reported a prevalence of 57.6% (95% CI = 56.9% to 58.3%).

**Table 2:** Subgroup Analyses of the Prevalence of Insomnia among Undergraduate Students Based on Random-Effect Analysis

Subgroup	**No. of reports**	**Prevalence (%)**	**95% CI**	***I*** ^**2**^ **(%)**	***P-*value for heterogeneity**	***P-*value between groups**
**Continent**						
Europe	10	52.8	46.7–58.9	96.7	<.001	<.001
Asia	30	49.4	38.1–60.7	99.8	<.001	
North America	11	33.3	25.9–40.6	99.3	<.001	
South America	2	44.1	42.1–46.2	–	–	
Africa	1	59.3	57.3–61.2	–	–	
Multiple	1	57.6	56.9–58.3	–	–	
**Field of study**						
Mixed samples	32	45.5	38.0–53.1	99.8	<.001	<.816
Medical	15	46.7	26.9–66.6	99.9	<.001	
Nursing	8	52.4	32.5–72.3	99.3	<.001	
**Assessment instrument**						
ISI	32	51.0	43.1–58.8	99.7	<.001	<.001
AIS	15	51.7	37.7–65.7	99.9	<.001	
SCI	5	28.5	21.1–35.9	98.5	<.001	
SLEEP-50	3	22.8	9.8–35.8	97.7	<.001	
**Sampling method**						
Non-probability	47	47.0	39.5–54.4	99.8	<.001	0.906
Probability	8	46.1	34.0–58.2	98.2	<.001	

CI: confidence intervals; *I*^2^: heterogeneity statistic (*I*-squared) expressed as a percentage. ISI: Insomnia Severity Index; AIS: Athens Insomnia Scale; SCI: Sleep Condition Indicator.

#### Screening instrument

Higher prevalence estimates were found in studies that used the AIS (51.7%; 95% CI = 37.7% to 65.7%) and the ISI (51.0%; 95% CI = 43.1% to 58.8%), compared with those that used the SCI (28.5%; 95% CI = 21.1% to 35.9%) and the SLEEP-50 (22.8%; 95% CI = 9.8% to 35.8%). However, the estimate for the SCI is derived from five data points across only two studies, and the SLEEP-50 is based on just three studies; therefore, these results should be interpreted with caution.

#### Field of study

The prevalence of insomnia in undergraduate nursing students was estimated from eight separate estimates derived from five citations. We observed heterogeneity in rates, from 18.0% (95% CI = 16.0–20.3) in Japan to 80.0% (95% CI = 70.6–87.0) in United Arab Emirates. The pooled prevalence of insomnia in undergraduate nursing students was 52.4% (95% CI = 32.5% to 72.3%). Among **medical students**, 15 citations were included. Prevalence estimates varied widely from 7.6% (95% CI = 6.1% to 9.6%) in China to 98.5% (95% CI = 96.7% to 99.3%) in Saudi Arabia. The overall pooled prevalence of insomnia in this group was 46.7% (95% CI = 26.9% to 66.6%). Lastly, the prevalence of insomnia in mixed samples of undergraduate students was estimated from 32 separate estimates derived from 28 citations. Estimates ranged from 11.6% (95% CI = 11.1% to 12.1%) in China to 74.5% (95% CI = 70.5% to 78.2%) in Saudi Arabia, with a pooled prevalence of 45.5% (95% CI = 38.0% to 53.1%).

#### Sampling method

Studies using non-probability sampling reported a pooled prevalence of 46.5% (95% CI = 39.2% to 53.8%), while those employing probability sampling reported a very similar rate of 46.2% (95% CI = 34.0% to 58.2%).

### Sensitivity analyses

Meta-regressions (see Table 3) indicated that samples from North America were significantly associated with lower insomnia prevalence estimates compared with all other continents (*β* = −0.770, 95% CI [−1.397 to −0.142], *p* = .016). Similarly, the use of the SCI (*β* = −0.894, 95% CI [−1.781 to −0.007], *p* = .048) and the SLEEP-50 (*β* = −1.211, 95% CI [−2.332 to −0.090], *p* = .034) was associated with significantly lower prevalence estimates relative to other measurement tools. We found no indication that other continents, field of study, study quality, sampling method, proportion of female participants, and publication year were significantly related to variation in prevalence estimates (all *p* ≥ .05). A leave-one-out sensitivity analysis was conducted by iteratively removing one study at a time, and no individual study altered the overall prevalence estimate by more than 1.5%.

**Table 3:** Results of Meta-Regression Analyses

Covariate	***β* (95% CI)**	**Standard error**	***P-*value**
**Year of publication**	0.077 (−0.016 to 0.170)	0.048	.105
**%Women**	1.090 (−0.765 to 2.945)	0.946	.249
**Risk of bias**	−0.139 (−0.425 to 0.147)	0.146	.341
**Other continents (Ref)**			
Europe	0.304 (−0.379 to 0.987)	0.348	.383
Asia	0.268 (−0.261 to 0.798)	0.270	.320
Africa	0.523 (−1.451 to 2.498)	1.007	.603
North America	**−0.770 (−1.397 to −0.142)**	**0.320**	**.016**
South America	−0.153 (−1.568 to 1.261)	0.721	.832
**Probability sampling**	−0.038 (−0.794 to 0.717)	0.385	.921
**Other instruments (Ref)**			
ISI	0.223 (−0.332 to 0.758)	0.273	.414
SCI	**−0.894 (−1.781 to −0.007)**	**0.452**	**.048**
AIS	.420 (−.169 to 1.009)	0.300	.162
SLEEP-50	**−1.211 (−2.332 to −0.090)**	**0.572**	**.034**
**Other degree (Ref)**			
Nursing	0.381 (−0.472 to 1.034)	0.384	.465
Medical	0.076 (−0.523 to 0.675)	0.306	.803
Mixed sample	−0.205 (−0.742 to 0.333)	0.274	.456

*Note:* Bold values indicate statistically significant results at p < .05; *β*: regression coefficient; CI: confidence intervals; Ref: reference; ISI: Insomnia Severity Index; AIS: Athens Insomnia Scale; SCI: Sleep Condition Indicator.

## Discussion

### Summary of findings

Our systematic review identified 48 studies yielding 55 separate prevalence estimates published between 2010 and 2024. The findings indicate nearly half of undergraduate university students worldwide experience symptoms of insomnia. We presented global- and regional-level data, as well as data based on the screening instruments used and field of study. Although we found no evidence that prevalence varied by study quality, field of study, sampling method, time period, or proportion of female participants, we observed evidence of lower prevalence in studies conducted in North America and in those using the SCI and SLEEP-50. Taken together with the high levels of statistical heterogeneity observed in our meta-analyses, these findings suggest methodological variation may partially obscure true differences in the prevalence of insomnia.

### Meaning of findings

Insomnia is a significant public health problem [1], and university students often face circumstances that are not conducive to healthy sleep patterns [11, 41]. It is therefore unsurprising that the prevalence of insomnia in this population is higher than in the general population, where the latest systematic review and meta-analysis reported a prevalence of 16.3% (95% CI = 11.3% to 23.0%) based on self-report questionnaires [42]. In contrast, our review indicates that ~46.9% of university students experience symptoms of insomnia, suggesting they are nearly three times as likely to be affected by this condition. More specifically, van Straten et al. reported prevalence estimates of 32.0% (95% CI = 22.1% to 44.6%) using the AIS, 25.1% (95% CI = 17.8% to 34.2%) based on the ISI with a cutoff of 8, and 12.5% (95% CI = 5.3% to 26.8%) using a cutoff of 10. In contrast, our findings show markedly higher rates among university students: 51.7% (95% CI = 37.7% to 65.7%) using the AIS, and 51.0% (95% CI = 43.1% to 58.8%) when applying ISI cutoffs of 8 and 10. Despite its high prevalence, poor sleep is often normalized among university students, which may hinder recognition of sleep problems and reduce help-seeking [43, 44]. Many students perceive sleep difficulties as a typical and temporary part of student life, often opting to self-manage rather than seek support, and may remain unaware of the potential long-term consequences of persistent sleep problems [44, 45].

Our pooled estimate of the global prevalence of insomnia in students (46.9% [95% CI = 40.1% to 53.6%]) is substantially higher than that reported in the previous systematic review by Jiang et al. [26], which estimated a prevalence of 18.5% [95% CI = 11.2% to 28.8%]. There are a number of plausible factors that may account for this discrepancy. First, there are differences in terms of the assessment of insomnia; while Jiang et al. included generalized sleep disturbance measures (e.g. Pittsburgh Sleep Quality Index) that lack specificity for insomnia, our review included only studies that employed validated self-report instruments specifically designed to assess insomnia. Second, their review included only seven studies, one of which was conducted in the general population and focused on individuals who had ever been students, rather than those who were students at the time their sleep difficulties were assessed. This study accounted for nearly 63% of the total sample and reported the lowest prevalence rate in their review. Third, the increasing academic, social, and psychological pressures faced by university students in recent years, particularly in the context of expanding digital demands and the COVID-19 pandemic, may have contributed to a genuine rise in insomnia prevalence over time.

The observed variation in reported prevalence estimates may be explained in part by differences in cultural and geographical context. We identified regional disparities in insomnia prevalence, with the highest rates observed in Europe (52.8%), followed by Asia (49.4%) and South America (44.1%), while North America reported the lowest prevalence at 33.3%. One plausible explanation for these geographical differences may be attributed to disparities in access to and utilization of mental health services [46], which may vary significantly across regions and may influence both the experience and reporting of insomnia symptoms. Additional contributing factors may include differing levels of academic pressure between countries or the influence of cultural norms and beliefs, which can shape how sleep difficulties are perceived and managed [47]. Variations in sleep health education and management for students may also play a role. Furthermore, the inclusion of international students in study samples may also act as a confounding factor, as their unique stressors, acculturative challenges, and culturally shaped perspectives may differ substantially from those of domestic students, further complicating cross-regional comparisons.

This variation may be also explained by differences in the cutoff scores used by each screening instrument to define insomnia. Studies employing the ISI or AIS reported higher prevalence rates compared to those using the SCI. In our meta-analysis, the ISI was scored using cutoffs of ≥8 and ≥ 10. A cutoff of ≥10 is widely recognized as optimal for detecting clinically significant insomnia in community samples and is commonly used in epidemiological research to identify insomnia cases in the general population. Where possible, this cutoff was utilized in the present analysis. A lower cutoff of ≥8 is often used to identify mild or subthreshold insomnia, while a cutoff of ≥15 has been proposed to indicate moderate and severe cases [48]. Similarly, the AIS used a cutoff score of ≥6 which is suggestive of mild or subthreshold insomnia according to the ISI classification described above [49]. In contrast, the SCI applies a cutoff of ≤16 and has demonstrated high specificity for detecting probable insomnia disorder, typically aligning with more severe cases as defined by an ISI score of ≥15 [40]. Therefore, the ISI and AIS are likely to capture a broader range of insomnia severity, including milder or more transient forms, whereas the SCI may be more selective for clinically significant insomnia. These differences in case identification thresholds likely contribute to the higher prevalence estimates observed in studies using the ISI or AIS. Taken together, our findings suggest that developing international guidelines for investigating the prevalence of insomnia could help minimize methodological heterogeneity in its reporting across the globe.

### Strengths and limitations

Strengths of our systematic review include that the methodology was registered prospectively, and the review was conducted in accordance with PRISMA guidelines. We conducted comprehensive searches across multiple databases without applying restrictions based on location or language. Furthermore, we included studies that specifically measured insomnia symptoms rather than general sleep quality, unlike previous meta-analyses [25, 26].

Nevertheless, our findings should be interpreted in the context of several limitations. First, the included studies relied on self-report questionnaires to assess insomnia. Such tools represent a balance between sensitivity and specificity and do not necessarily confirm a clinical diagnosis of insomnia disorder, which requires a structured clinical assessment. As such, our prevalence estimates may reflect elevated symptoms rather than true disorder rates. With reference to the last point, the sample may have also contained a significant number of individuals with a circadian delay, affecting the prevalence rates reported. As is well reported in the literature, the post-adolescent phase of development commonly presents with a circadian delay and this can mimic one of the main symptoms of insomnia (i.e. increased sleep latency) [50, 51]. While this is usually considered in clinical interviews, this is not necessarily the case for questionnaires. As such, we strongly suggest future research take account of this with circadian specific questions and circadian specific questionnaires to disentangle this relationship. Further, although pooling data from different questionnaires is a common approach in meta-analytic research, it remains a potentially problematic practice that may introduce bias into the results [52]. In our subgroup analysis, we observed that prevalence estimates varied depending on the instrument used. While these differences may reflect real differences in insomnia rates, they are more likely attributable to measurement inconsistencies, which could, in turn, compromise the validity of our findings. Second, most included studies had disproportionately higher participation from female students. Given that the prevalence of insomnia is significantly higher among females than males [5], this imbalance may have contributed to the higher overall prevalence observed across studies and in the pooled results. Third, most studies used convenience sampling methods and had unclear or relatively modest response rates, which may have introduced biases, potentially leading to an overestimation (or underestimation) of prevalence estimates. This is perhaps unsurprising, considering that much of the research conducted among university students has been limited by small, non-representative samples, with probability-based sampling methods rarely employed. However, our meta-regression analysis revealed no significant differences in prevalence rates between studies using probability-based sampling and those using non-probability methods. This suggests either that the sampling method did not significantly influence the overall prevalence estimates, or that our analysis lacked sufficient statistical power to detect such an effect. Fourth, the majority of included studies were conducted in Asia, Europe, and North America, which limits the geographical representativeness of our findings. Only one study was conducted in Africa, and none originated from Australia, potentially overlooking regional and environmental factors that may influence the prevalence of insomnia. Fifth, there was substantial heterogeneity across studies, which remained largely unexplained despite subgroup and meta-regression analyses. It is possible that exploring heterogeneity across intersecting moderator variables, such as academic pressure, cultural attitudes toward sleep, or access to mental health resources, could provide further insight; however, this level of analysis was beyond the scope of the present study.

### Implications for practice, policy, and research

Despite the aforementioned limitations, our review provides evidence that a substantial proportion of undergraduate university students experience insomnia symptoms. While universities are not primarily responsible for delivering clinical care, community mental health services often face challenges in meeting the needs of a large, diverse, and transient student population. Universities are therefore well positioned to adopt a proactive, comprehensive, and evidence-based approach to support student mental health, including insomnia [53]. A two-level intervention strategy is recommended. At the population level, universities should implement public health initiatives such as the widespread dissemination of evidence-based sleep guidance, the integration of digital sleep hygiene modules, and the inclusion of sleep education within existing wellbeing programs. At the individual level, evidence-based interventions, such as digital Cognitive Behavioral Therapy for Insomnia (CBT-I), could be embedded within university support services. CBT-I is unequivocally recognized as the first-line treatment for insomnia [54, 55] and has demonstrated robust effectiveness in reducing insomnia symptom severity across various settings and populations [56–58], including university students [59]. The diverse ways in which CBT-I can be delivered (e.g. individual face-to-face, group therapy, using telehealth or digitally) has the potential to improve treatment access and address the needs of students experiencing sleep difficulties at all levels of complexity and need [60].

Sleep promotion and intervention strategies integrated into the infrastructure of higher education have the potential to reduce the prevalence and severity of insomnia, enhance academic performance, and improve overall student health. Moreover, early identification and treatment of insomnia may serve as a preventative measure, mitigating sleep-related challenges and lowering the risk of developing future mental health difficulties. This is particularly important given the established role of insomnia as a predictor of future psychopathology, including anxiety and depression [18]. Evidence also suggests that treating sleep difficulties can lead to improvements in comorbid mental health symptoms, both among university students [61] and in clinical populations [62]. Further research findings indicate a dose–response relationship, whereby greater improvements in sleep quality are associated with more substantial improvements in mental health outcomes [63]. Taken together, these findings highlight the importance of addressing sleep problems early and systematically as a cost-effective and scalable strategy to enhance both student mental health and academic success.

Globally, future research should address methodological limitations to improve the reliability and generalizability of findings. This includes the use of probability sampling methods, larger and more diverse sample sizes, and transparent reporting of response rates to reduce potential biases and improve representativeness. Additionally, there is a clear need for more studies from underrepresented regions, such as Africa, Australia, and South America, to better understand cross-cultural differences in insomnia prevalence among university students. Lastly, rigorous evaluation of interventions specifically tailored to student populations is essential. In particular, scalable approaches such as low-intensity digital interventions should be examined to determine their long-term effectiveness and feasibility within higher education settings.

## Conclusion

The present study indicated that nearly half of undergraduate university students worldwide experience symptoms of insomnia, with prevalence estimates varying by geographic region and screening instruments used. These findings highlight the need for university-based responses that integrate universal sleep health promotion with targeted interventions for students experiencing more severe or persistent symptoms. Further methodologically rigorous and culturally appropriate epidemiological research is needed to better understand and address the global burden of insomnia in this population.

## Supplementary Material

Supplementary_materials_zpaf083

## Data Availability

The data that support the findings of this study are available from the corresponding author upon reasonable request.
