# Supplementary material for: Global prevalence of insomnia symptoms in undergraduate university students: a systematic review and meta-analysis
Source: Sleep Adv. 2025 Nov 17;6(4):zpaf083. doi: 10.1093/sleepadvances/zpaf083 (PMC12687938; doi:10.1093/sleepadvances/zpaf083)
Supplement: Supplementary_materials_zpaf083 [file supplementary_materials_zpaf083.pdf]

TITLE PAGE

Global prevalence of insomnia symptoms in undergraduate university students: A systematic review and meta-analysis

Authors

Spyros Spyridonidis[1,2*], Dhillon Lad[1,2], Harrison Peters[1,2], Jason Ellis[3], Lucy J Robinson[1]

[1] Newcastle University, Newcastle upon Tyne, United Kingdom

[2] Cumbria, Northumberland, Tyne & Wear NHS Foundation Trust, Newcastle upon Tyne, United Kingdom

[3] Northumbria University, Newcastle upon Tyne, United Kingdom

***Correspondence to:** Spyros Spyridonidis, School of Psychology, Newcastle University, Wallace Street, Newcastle upon Tyne, NE2 4DR, email: s.spyridonidis2@newcastle.ac.uk

Table S1 PRISMA checklist

Section and Topic	Item #	Checklist item	Location where item is reported
**TITLE**			
Title	1	Identify the report as a systematic review.	1
**ABSTRACT**			
Abstract	2	See the PRISMA 2020 for Abstracts checklist.	2
**INTRODUCTION**			
Rationale	3	Describe the rationale for the review in the context of existing knowledge.	Introduction
Objectives	4	Provide an explicit statement of the objective(s) or question(s) the review addresses.	Introduction
**METHODS**			
Eligibility criteria	5	Specify the inclusion and exclusion criteria for the review and how studies were grouped for the syntheses.	Methods: Eligibility criteria
Information sources	6	Specify all databases, registers, websites, organisations, reference lists and other sources searched or consulted to identify studies. Specify the date when each source was last searched or consulted.	Methods: Search strategy. Tables S2-S6
Search strategy	7	Present the full search strategies for all databases, registers and websites, including any filters and limits used.	Tables S2-S6
Selection process	8	Specify the methods used to decide whether a study met the inclusion criteria of the review, including how many reviewers screened each record and each report retrieved, whether they worked independently, and if applicable, details of automation tools used in the process.	Methods: Eligibility criteria, study selection
Data collection process	9	Specify the methods used to collect data from reports, including how many reviewers collected data from each report, whether they worked independently, any processes for obtaining or confirming data from study investigators, and if applicable, details of automation tools used in the process.	Methods: Data extraction
Data items	10a	List and define all outcomes for which data were sought. Specify whether all results that were compatible with each outcome domain in each study were sought (e.g. for all measures, time points, analyses), and if not, the methods used to decide which results to collect.	Methods: Data extraction
	10b	List and define all other variables for which data were sought (e.g. participant and intervention characteristics, funding sources). Describe any assumptions made about any missing or unclear information.	Methods: Data extraction
Study risk of bias assessment	11	Specify the methods used to assess risk of bias in the included studies, including details of the tool(s) used, how many reviewers assessed each study and whether they worked independently, and if applicable, details of automation tools used in the process.	Methods: Risk of bias assessment
Effect measures	12	Specify for each outcome the effect measure(s) (e.g. risk ratio, mean difference) used in the synthesis or presentation of results.	Methods: Data synthesis

Section and Topic	Item #	Checklist item	Location where item is reported
Synthesis methods	13a	Describe the processes used to decide which studies were eligible for each synthesis (e.g. tabulating the study intervention characteristics and comparing against the planned groups for each synthesis (item #5)).	Methods: Eligibility criteria, Data synthesis
	13b	Describe any methods required to prepare the data for presentation or synthesis, such as handling of missing summary statistics, or data conversions.	Methods: Data extraction
	13c	Describe any methods used to tabulate or visually display results of individual studies and syntheses.	Methods: Data synthesis
	13d	Describe any methods used to synthesize results and provide a rationale for the choice(s). If meta-analysis was performed, describe the model(s), method(s) to identify the presence and extent of statistical heterogeneity, and software package(s) used.	Methods: Data synthesis
	13e	Describe any methods used to explore possible causes of heterogeneity among study results (e.g. subgroup analysis, meta-regression).	Methods: Data synthesis
	13f	Describe any sensitivity analyses conducted to assess robustness of the synthesized results.	Methods: Data synthesis
Reporting bias assessment	14	Describe any methods used to assess risk of bias due to missing results in a synthesis (arising from reporting biases).	Methods: Risk of bias assessment, Data synthesis
Certainty assessment	15	Describe any methods used to assess certainty (or confidence) in the body of evidence for an outcome.	Methods: Data synthesis
**RESULTS**			
Study selection	16a	Describe the results of the search and selection process, from the number of records identified in the search to the number of studies included in the review, ideally using a flow diagram.	Figure 1
	16b	Cite studies that might appear to meet the inclusion criteria, but which were excluded, and explain why they were excluded.	Figure 1
Study characteristics	17	Cite each included study and present its characteristics.	Table 1
Risk of bias in studies	18	Present assessments of risk of bias for each included study.	Table S7
Results of individual studies	19	For all outcomes, present, for each study: (a) summary statistics for each group (where appropriate) and (b) an effect estimate and its precision (e.g. confidence/credible interval), ideally using structured tables or plots.	Results, Figure 2
Results of syntheses	20a	For each synthesis, briefly summarise the characteristics and risk of bias among contributing studies.	Results: Study characteristics, Table S7

Section and Topic	Item #	Checklist item	Location where item is reported
	20b	Present results of all statistical syntheses conducted. If meta-analysis was done, present for each the summary estimate and its precision (e.g. confidence/credible interval) and measures of statistical heterogeneity. If comparing groups, describe the direction of the effect.	Results
	20c	Present results of all investigations of possible causes of heterogeneity among study results.	Results: Subgroup analyses, Sensitivity analyses
	20d	Present results of all sensitivity analyses conducted to assess the robustness of the synthesized results.	Results: Sensitivity analyses
Reporting biases	21	Present assessments of risk of bias due to missing results (arising from reporting biases) for each synthesis assessed.	Not reported
Certainty of evidence	22	Present assessments of certainty (or confidence) in the body of evidence for each outcome assessed.	Results, Tables 2,3
**DISCUSSION**			
Discussion	23a	Provide a general interpretation of the results in the context of other evidence.	Discussion, Meaning of findings
	23b	Discuss any limitations of the evidence included in the review.	Discussion: Strengths and limitations
	23c	Discuss any limitations of the review processes used.	Discussion: Strengths and limitations
	23d	Discuss implications of the results for practice, policy, and future research.	Discussion, Conclusions
**OTHER INFORMATION**			
Registration and protocol	24a	Provide registration information for the review, including register name and registration number, or state that the review was not registered.	Methods
	24b	Indicate where the review protocol can be accessed, or state that a protocol was not prepared.	Methods
	24c	Describe and explain any amendments to information provided at registration or in the protocol.	Methods
Support	25	Describe sources of financial or non-financial support for the review, and the role of the funders or sponsors in the review.	Disclosure statement
Competing interests	26	Declare any competing interests of review authors.	Disclosure statement

Section and Topic	Item #	Checklist item	Location where item is reported
Availability of data, code and other materials	27	Report which of the following are publicly available and where they can be found: template data collection forms; data extracted from included studies; data used for all analyses; analytic code; any other materials used in the review.	Data availability statement

From: Page MJ, McKenzie JE, Bossuyt PM, Boutron I, Hoffmann TC, Mulrow CD, et al. The PRISMA 2020 statement: an updated guideline for reporting systematic reviews. BMJ 2021;372:n71. doi: 10.1136/bmj.n71

For more information, visit: http://www.prisma-statement.org/

Supplementary Table 2. EMBASE via OVID (up to Jan 17, 2025)

#	**Advanced searches**	**Results**
1.	exp insomnia/	92076
2.	insomnia*.mp.	100914
3.	"Sleep Initiation and Maintenance Disorder*".mp.	431
4.	1 or 2 or 3	101544
5.	(university student* or college student* or undergraduate* or postgraduate* or graduate*).mp.	230868
6.	(prevalen* or rate* or epidemiolog* or frequency).mp.	8788657
7.	4 and 5 and 6	806
8.	limit 7 to human	793
9.	limit 8 to yr="1993 - 2025"	793

Supplementary Table 3. Web of Science (up to Jan 17, 2025)

#	**Advanced searches**	**Results**
1.	((ALL=(insomnia* OR "Sleep Initiation and Maintenance Disorder*")) AND ALL=(university student* OR college student* OR undergraduate* OR postgraduate* OR graduate*)) AND ALL=(prevalen* OR rate* OR epidemiolog* OR frequency)<br>Publication date: 01/01/1993 – 17/01/2025	1439

Supplementary Table 4. Ovid MEDLINE(R) and Epub Ahead of Print, In-Process, In-Data-Review & Other Non-Indexed Citations, Daily and Versions (up to Jan 17, 2025)

#	**Advanced searches**	**Results**
1.	exp insomnia/	19737
2.	insomnia*.mp.	34334
3.	"Sleep Initiation and Maintenance Disorder*".mp.	19685
4.	1 or 2 or 3	39530
5.	(university student* or college student* or undergraduate* or postgraduate* or graduate*).mp.	237602
6.	(prevalen* or rate* or epidemiolog* or frequency).mp.	7279884
7.	4 and 5 and 6	471
8.	limit 7 to humans	382

Supplementary Table 5. CINAHL via EBSCOhost (up to Jan 17, 2025)

#	Search options	Actions	Results
S15	S3 AND S9 AND S14	**Limits: 1993 -2025**<br>**Search modes** - Boolean/Phrase	391
S14	S10 OR S11 OR S12 OR S13	**Limits: 1993 -2025**<br>**Search modes** - Boolean/Phrase	1702536
S13	TX "Epidemiolog*"	**Limits: 1993 -2025**<br>**Search modes** - Boolean/Phrase	751215
S12	TX "Frequen*"	**Limits: 1993 -2025**<br>**Search modes** - Boolean/Phrase	380182
S11	TX "Rate*"	**Limits: 1993 -2025**<br>**Search modes** - Boolean/Phrase	730056
S10	TX "Prevalen*"	**Limits: 1993 -2025**<br>**Search modes** - Boolean/Phrase	322636
S9	S4 OR S5 OR S6 OR S7 OR S8	**Limits: 1993 -2025**<br>**Search modes** - Boolean/Phrase	299072
S8	TX "Graduate*"	**Limits: 1993 -2025**<br>**Search modes** - Boolean/Phrase	203837
S7	TX "Postgraduate*"	**Limits: 1993 -2025**<br>**Search modes** - Boolean/Phrase	43492
S6	TX "Undergraduate*"	**Limits: 1993 -2025**<br>**Search modes** - Boolean/Phrase	41321
S5	TX "University student*"	**Limits: 1993 -2025**<br>**Search modes** - Boolean/Phrase	11181
S4	TX "College student*"	**Limits: 1993 -2025**<br>**Search modes** - Boolean/Phrase	17738
S3	S1 OR S2	**Limits: 1993 -2025**<br>**Search modes** - Boolean/Phrase	15514
S2	TX "Sleep Initiation and Maintenance Disorder*"	**Limits: 1993 -2025**<br>**Search modes** - Boolean/Phrase	13
S1	TX "Insomnia*"	**Limits: 1993 -2025**<br>**Search modes** - Boolean/Phrase	15510

Supplementary Table 6. PsycIFNO via Ovid (up to Jan 17, 2025)

#	Search options	Results
1.	insomnia*.mp.	17545
2.	exp insomnia/	8916

3.	"Sleep Initiation and Maintenance Disorder*".mp.	4929
4.	1 or 2 or 3	18636
5.	(university student* or college student* or undergraduate* or postgraduate* or graduate*).mp.	266978
6.	(prevalen* or rate* or epidemiolog* or frequency).mp.	803692
7.	4 and 5 and 6	229
8.	limit 7 to human	223
9.	limit 8 to yr="1993 - 2025"	222

Hand search

Please note that the hand search list was comprised of the 48 included studies and 3 reviews noted in the main text. Five studies were identified by hand search (see bolded articles below), and then the references of these five studies were also checked.

Alshammari et al. (2023), Eleftheriou et al. (2021), Gaultney (2010), Ramon-Arbues et al. (2020), and Shakeel et al. (2019)

Supplementary Table 7. Excluded studies with reason (N = 366)

#	Citation	Reason
1	Abdel Khalek et al. (1995)	Did not report outcome of interest
2	Abdel-Khalek (2006)	Used improper outcome measure
3	Abdel-Khalek (2004)	Had unrelated population
4	Abdel-Khalek (2008)	Did not report outcome of interest
5	Abdulah and Piro (2018)	Did not report outcome of interest
6	Ahmed et al. (2022)	Used improper outcome measure
7	Ahmed et al. (2021)	Had unrelated population
8	Ahrberg et al. (2012)	Did not meet the eligible population criteria
9	Akram et al. (2019)	Did not meet the eligible population criteria
10	Al Salmani et al. (2020)	Did not meet the eligible population criteria
11	Al-Mamun et al. (2024)	Had unrelated population
12	Alageel et al. (2021)	Did not meet the eligible population criteria
13	Albasheer et al. (2020)	Did not meet the eligible population criteria
14	Albikawi (2023)	Did not meet the eligible population criteria
15	Aldrin Joshua et al. (2020)	Did not meet the eligible population criteria
16	Alfadeel et al. (2019)	Did not meet the eligible population criteria
17	Alfawaz et al. (2021)	Did not meet the eligible population criteria
18	AlHadi and Alhuwaydi (2022)	Did not meet the eligible population criteria
19	Alkahtani et al. (2022)	Had unrelated population
20	Almansour et al. (2020)	Did not report outcome of interest
21	Almusa (2021)	Had unrelated population
22	Alomri and Alghamdi (2024)	Did not meet the eligible population criteria
23	Alqahtani et al. (2022)	Had unrelated population
24	Alsaadi (2022)	Did not report outcome of interest
25	Alshammari et al. (2024)	Did not meet the eligible population criteria
26	Alsuhaymi et al. (2019)	Did not meet the eligible population criteria
27	Alyoubi et al. (2021)	Did not report outcome of interest
28	Amaral et al. (2018)	Did not report outcome of interest
29	Amelia et al. (2023)	Did not meet the eligible population criteria
30	Amlak et al. (2022)	Did not report outcome of interest
31	Andorko et al. (2017)	Used improper outcome measure
32	Angelone et al. (2011)	Used improper outcome measure
33	Anupama et al. (2022)	Did not meet the eligible population criteria
34	Anwer et al. (2021)	Did not meet the eligible population criteria
35	Apaza-Panca et al. (2021)	Did not report outcome of interest
36	Araujo et al. (2014)	Did not report outcome of interest
37	Arbinaga et al. (2019)	Did not report outcome of interest
38	Assefa et al. (2021)	Did not report outcome of interest

**39**	Atlam and Elsabagh (2020)	Used improper outcome measure
**40**	Baalaraj et al. (2022)	Used improper outcome measure
**41**	Babicki et al. (2022)	Overlap data
**42**	Backovic et al. (2013)	Used improper outcome measure
**43**	Badellino et al. (2024)	Full-text unavailable
**44**	Baker et al. (2015)	Did not meet the eligible population criteria
**45**	Bastien et al. (2019)	Did not report outcome of interest
**46**	Baylor et al. (2016)	Used improper outcome measure
**47**	Becker et al. (2018)	Did not meet the eligible population criteria
**48**	Belingheri et al. (2022)	Used improper outcome measure
**49**	Belingheri et al. (2020)	Did not report outcome of interest
**50**	Benham et al. (2019)	Did not report outcome of interest
**51**	Benham (2022)	Did not report outcome of interest
**52**	Benham (2021)	Did not report outcome of interest
**53**	Bhakat and Das (2023)	Did not report outcome of interest
**54**	Brown et al. (2002)	Did not meet the eligible population criteria
**55**	Brunk (2019)	Did not meet the eligible population criteria
**56**	Buboltz et al. (2001)	Used improper outcome measure
**57**	Buckner et al. (2008)	Did not report outcome of interest
**58**	Calderon et al. (2024)	Did not report outcome of interest
**59**	Carney et al. (2006)	Did not report outcome of interest
**60**	Carpi, Marques, et al. (2022)	Did not meet the eligible population criteria
**61**	Carpi, Cianfarani, et al. (2022)	Did not meet the eligible population criteria
**62**	Carrillo Ibarra et al. (2010)	Used improper outcome measure
**63**	Carrión-Pantoja et al. (2022)	Did not meet the eligible population criteria
**64**	Castaldelli-Maia et al. (2019)	Did not report outcome of interest
**65**	Castelnuovo et al. (2021)	Full-text unavailable
**66**	Cebrino and Portero de la Cruz (2023)	Did not report outcome of interest
**67**	Celik et al. (2019)	Did not report outcome of interest
**68**	Chan-Chee et al. (2011)	Had unrelated population
**69**	Chandler et al. (2023)	Did not meet the eligible population criteria
**70**	Chen et al. (2023)	Did not meet the eligible population criteria
**71**	M. Chen et al. (2024)	Did not meet the eligible population criteria
**72**	S. Chen et al. (2024)	Did not report outcome of interest
**73**	Cheng et al. (2023)	Did not meet the eligible population criteria
**74**	Cheng et al. (2012)	Used improper outcome measure
**75**	Cheraghi and Shamsaei (2008)	Full-text unavailable
**76**	Choueiry et al. (2016)	Did not meet the eligible population criteria
**77**	Chowdhury et al. (2024)	Did not meet the eligible population criteria
**78**	Christodoulou et al. (2023)	Did not report outcome of interest
**79**	Chu et al. (2016)	Did not report outcome of interest

**80**	Coelho et al. (2023)	Used improper outcome measure
**81**	Copaja-Corzo et al. (2022)	Used improper outcome measure
**82**	Correa Rangel et al. (2022)	Did not meet the eligible population criteria
**83**	Correa Rangel et al. (2021)	Did not meet the eligible population criteria
**84**	da Silva Cardoso et al. (2022)	Did not meet the eligible population criteria
**85**	da Silva-Fonseca et al. (2021)	Did not meet the eligible population criteria
**86**	Daghigh (2024)	Did not meet the eligible population criteria
**87**	Dagnew et al. (2020)	Did not report outcome of interest
**88**	De Souza (1996)	Did not meet the eligible population criteria
**89**	Deng et al. (2023)	Did not report outcome of interest
**90**	Dewan et al. (2022)	Did not meet the eligible population criteria
**91**	Denis et al. (2019)	Did not report outcome of interest
**92**	Doos Ali Vand et al. (2014)	Did not report outcome of interest
**93**	Duan et al. (2022)	Used improper outcome measure
**94**	El Ansari et al. (2014)	Did not report outcome of interest
**95**	El Hangouche et al. (2018)	Did not report outcome of interest
**96**	El Sahly et al. (2020)	Used improper outcome measure
**97**	El-Anzi (2006)	Did not report outcome of interest
**98**	Ergin et al. (2022)	Did not report outcome of interest
**99**	Etindele-Sosso (2020)	Had unrelated population
**100**	Eva et al. (2024)	Did not report outcome of interest
**101**	Falavigna et al. (2011)	Used improper outcome measure
**102**	Faria et al. (2014)	Full-text unavailable
**103**	Feher et al. (2023)	Had unrelated population
**104**	Fernandes et al. (2022)	Did not meet the eligible population criteria
**105**	Fernandez-Mendoza et al. (2009)	Used improper outcome measure
**106**	Fila-Witecka et al. (2021)	Did not report outcome of interest
**107**	Floyd and Vargas (2024)	Did not meet the eligible population criteria
**108**	Foster et al. (2023)	Did not report outcome of interest
**109**	Friedrich et al. (2016)	Used improper outcome measure
**110**	Gao, Wang, Liu, Wang, Song, et al. (2024)	Did not meet the eligible population criteria
**111**	Gao, Wang, Liu, Wang, Xiong, et al. (2024)	Did not meet the eligible population criteria
**112**	Gaultney et al. (2012)	Did not report outcome of interest
**113**	Gellis et al. (2014)	Did not meet the eligible population criteria
**114**	Gellis (2011)	Full-text unavailable
**115**	Gemnani et al. (2019)	Did not meet the eligible population criteria
**116**	Ghrouz et al. (2021)	Used improper outcome measure
**117**	Ghrouz et al. (2019)	Did not report outcome of interest
**118**	Gianfredi et al. (2018)	Did not meet the eligible population criteria
**119**	Gilstrap et al. (2023)	Did not meet the eligible population criteria
**120**	Goel et al. (2023)	Did not meet the eligible population criteria

**121**	Goel et al. (2021)	Did not meet the eligible population criteria
**122**	Gonçalves et al. (2017)	Did not report outcome of interest
**123**	Goodhines et al. (2019)	Did not meet the eligible population criteria
**124**	Goodhines et al. (2016)	Full-text unavailable
**125**	Gorgich et al. (2018)	Did not report outcome of interest
**126**	Gruezo-Realpe, Benavides-Lopez, et al. (2023)	Did not meet the eligible population criteria
**127**	Gruezo-Realpe, Chango-Pinargote, et al. (2023)	Did not meet the eligible population criteria
**128**	Günes and Arslantas (2017)	Did not report outcome of interest
**129**	Guo et al. (2014)	Had unrelated population
**130**	Guo et al. (2023)	Did not meet the eligible population criteria
**131**	Gupta et al. (2021)	Did not meet the eligible population criteria
**132**	Gupta et al. (2018)	Did not meet the eligible population criteria
**133**	Haghighi and Gerber (2019)	Did not meet the eligible population criteria
**134**	Haile et al. (2017)	Used improper outcome measure
**135**	Hall et al. (2017)	Did not meet the eligible population criteria
**136**	Hamilton et al. (2019)	Had unrelated population
**137**	Hammoudi et al. (2021)	Did not meet the eligible population criteria
**138**	Hanna and Carter (2013)	Did not meet the eligible population criteria
**139**	Hartmann and Prichard (2018)	Did not report outcome of interest
**140**	Hayer and Hicks (1993)	Did not report outcome of interest
**141**	Hayley, Downey, et al. (2017)	Did not report outcome of interest
**142**	Hayley, Sivertsen, et al. (2017)	Did not report outcome of interest
**143**	Hendershot et al. (2021)	Full-text unavailable
**144**	Hershner et al. (2021)	Did not meet the eligible population criteria
**145**	Hidalgo and Caumo (2002)	Used improper outcome measure
**146**	Hjetland et al. (2021)	Used improper outcome measure
**147**	Hood et al. (2011)	Did not report outcome of interest
**148**	Hou (2021)	Did not report outcome of interest
**149**	Hsu and Chang (2022)	Did not meet the eligible population criteria
**150**	Hussein et al. (2012)	Did not meet the eligible population criteria
**151**	Ibrahim et al. (2024)	Did not meet the eligible population criteria
**152**	Ionescu et al. (2023)	Did not report outcome of interest
**153**	Jain et al. (2020)	Did not meet the eligible population criteria
**154**	Jones et al. (2019)	Used improper outcome measure
**155**	Jose et al. (2024)	Did not meet the eligible population criteria
**156**	Joshi et al. (2015)	Did not meet the eligible population criteria
**157**	Kadam et al. (2016)	Did not report outcome of interest
**158**	Kandeger and Selvi (2017)	Full-text unavailable
**159**	Kang et al. (2020)	Did not report outcome of interest
**160**	Kayaba et al. (2020)	Did not meet the eligible population criteria
**161**	Khader et al. (2020)	Did not report outcome of interest

**162**	Khalil et al. (2023)	Full-text unavailable
**163**	Khan et al. (2019)	Did not meet the eligible population criteria
**164**	Khan et al. (2024)	Did not meet the eligible population criteria
**165**	Kim et al. (2022)	Did not report outcome of interest
**166**	King et al. (2018)	Full-text unavailable
**167**	King et al. (2022)	Did not report outcome of interest
**168**	King, Pickett, et al. (2023)	Overlap data
**169**	King, Rivera, et al. (2023)	Overlap data
**170**	Kivela et al. (2024)	Did not report outcome of interest
**171**	Klainberg et al. (2021)	Did not report outcome of interest
**172**	Kwok et al. (2021)	Did not meet the eligible population criteria
**173**	Labiano and Fiorentino (1996)	Did not report outcome of interest
**174**	Lai et al. (2020)	Did not meet the eligible population criteria
**175**	Larrabee and Prichard (2016)	Full-text unavailable
**176**	Lawal et al. (2023)	Did not report outcome of interest
**177**	Lemma et al. (2012)	Did not report outcome of interest
**178**	Li and Xiao (2024)	Did not meet the eligible population criteria
**179**	Li et al. (2023)	Did not meet the eligible population criteria
**180**	F. Li (2024)	Did not meet the eligible population criteria
**181**	H. Li et al. (2020)	Did not report outcome of interest
**182**	J. Li et al. (2024)	Did not report outcome of interest
**183**	J. Li (2024)	Did not report outcome of interest
**184**	L. Li et al. (2024)	Did not report outcome of interest
**185**	Q. Li et al. (2024)	Used improper outcome measure
**186**	Li et al. (2021)	Had unrelated population
**187**	T. T. Li et al. (2020)	Did not report outcome of interest
**188**	Y. Li et al. (2020)	Did not report outcome of interest
**189**	Li et al. (2022)	Did not report outcome of interest
**190**	S.-w. Liang et al. (2022)	Did not report outcome of interest
**191**	Liang et al. (2025)	Did not meet the eligible population criteria
**192**	Y. Liang et al. (2022)	Did not report outcome of interest
**193**	Liao et al. (2024)	Did not report outcome of interest
**194**	Lindsay et al. (2022)	Overlap data
**195**	Liu et al. (2019)	Did not meet the eligible population criteria
**196**	Liu et al. (1995)	Full-text unavailable
**197**	Liu et al. (2000)	Had unrelated population
**198**	Liu et al. (2023)	Did not report outcome of interest
**199**	Liu et al. (2021)	Did not report outcome of interest
**200**	Loayza H et al. (2001)	Used improper outcome measure
**201**	Lohsoonthorn et al. (2013)	Did not report outcome of interest
**202**	Loke et al. (2022)	Full-text unavailable

**203**	Lombardo et al. (2014)	Did not meet the eligible population criteria
**204**	Lombardo (2012)	Had unrelated population
**205**	Low et al. (2024)	Did not meet the eligible population criteria
**206**	Lukowski et al. (2024)	Did not meet the eligible population criteria
**207**	Lukowski and Tsukerman (2021)	Did not meet the eligible population criteria
**208**	Lund et al. (2010)	Did not report outcome of interest
**209**	S. Ma et al. (2022)	Did not report outcome of interest
**210**	Z. Ma et al. (2022)	Used improper outcome measure
**211**	Madsen et al. (2023)	Did not meet the eligible population criteria
**212**	Mahfouz et al. (2020)	Did not report outcome of interest
**213**	Makhal et al. (2015)	Did not meet the eligible population criteria
**214**	Mansour and Yousef (2016)	Did not meet the eligible population criteria
**215**	Manzar (2020)	Used improper outcome measure
**216**	Manzar et al. (2019)	Did not report outcome of interest
**217**	Manzar et al. (2021)	Did not meet the eligible population criteria
**218**	Manzar et al. (2020)	Did not meet the eligible population criteria
**219**	Marta et al. (2020)	Did not meet the eligible population criteria
**220**	Matsuda and Kikutani (2022)	Did not meet the eligible population criteria
**221**	Mbous et al. (2022)	Did not meet the eligible population criteria
**222**	Meaklim et al. (2024)	Did not report outcome of interest
**223**	Meaklim et al. (2023)	Did not report outcome of interest
**224**	Meaklim et al. (2021)	Did not report outcome of interest
**225**	Medina-Ortiz et al. (2024)	Full-text unavailable
**226**	Mendoza et al. (2021)	Did not meet the eligible population criteria
**227**	Meng et al. (2021)	Did not report outcome of interest
**228**	Merchan Tamayo et al. (2024)	Did not report outcome of interest
**229**	Meyer-Szary et al. (2008)	Did not report outcome of interest
**230**	Miadich and Tagler (2011)	Full-text unavailable
**231**	Michaeli et al. (2022)	Used improper outcome measure
**232**	Milicev et al. (2023)	Did not meet the eligible population criteria
**233**	Miller et al. (2019)	Did not meet the eligible population criteria
**234**	Milojevich and Lukowski (2016)	Did not meet the eligible population criteria
**235**	Moayedi et al. (2015)	Did not meet the eligible population criteria
**236**	Moghaddam et al. (2012)	Used improper outcome measure
**237**	Mohamed et al. (2020)	Did not meet the eligible population criteria
**238**	Mohammadbeigi et al. (2016)	Did not report outcome of interest
**239**	Moked et al. (2016)	Full-text unavailable
**240**	Moo Estrella et al. (2013)	Did not meet the eligible population criteria
**241**	Moo-Estrella et al. (2005)	Did not report outcome of interest
**242**	Mousavi et al. (2022)	Did not meet the eligible population criteria
**243**	Moussa-Chamari, Farooq, et al. (2024)	Did not report outcome of interest

**244**	Moussa-Chamari, Romdhani, et al. (2024)	Did not meet the eligible population criteria
**245**	Mozaffari (2016)	Full-text unavailable
**246**	Muanprasong and Taneepanichskul (2017)	Did not report outcome of interest
**247**	Munezawa et al. (2009)	Had unrelated population
**248**	Nadeem et al. (2018)	Did not report outcome of interest
**249**	Michael R. Nadorff et al. (2013)	Did not report outcome of interest
**250**	Nadorff et al. (2014)	Did not report outcome of interest
**251**	M. R. Nadorff et al. (2013)	Full-text unavailable
**252**	Nagasaki et al. (2024)	Had unrelated population
**253**	Nagose et al. (2021)	Did not report outcome of interest
**254**	Nam et al. (2021)	Did not meet the eligible population criteria
**255**	Navarro-Martinez et al. (2020)	Did not meet the eligible population criteria
**256**	Nikolaev et al. (2024)	Full-text unavailable
**257**	Nisarga et al. (2018)	Did not report outcome of interest
**258**	Nojomi et al. (2009)	Used improper outcome measure
**259**	Nsengimana et al. (2023)	Did not report outcome of interest
**260**	Nugent et al. (2020)	Did not report outcome of interest
**261**	Oliveira et al. (2024)	Did not meet the eligible population criteria
**262**	Pacella et al. (2024)	Did not meet the eligible population criteria
**263**	Pallesen et al. (2008)	Did not report outcome of interest
**264**	Paudel et al. (2024)	Did not meet the eligible population criteria
**265**	Pavlova and Rogowska (2023)	Did not meet the eligible population criteria
**266**	Pervez et al. (2021)	Did not meet the eligible population criteria
**267**	Petrie et al. (2023)	Did not report outcome of interest
**268**	Petrov et al. (2014)	Used improper outcome measure
**269**	Piotrowski et al. (2021)	Did not meet the eligible population criteria
**270**	Poorolajal et al. (2017)	Did not report outcome of interest
**271**	Powell and Nielsen (2019)	Did not report outcome of interest
**272**	Qeadan et al. (2024)	Did not report outcome of interest
**273**	Qiao et al. (2023)	Used improper outcome measure
**274**	Ramirez et al. (2022)	Full-text unavailable
**275**	Ramón-Arbués et al. (2022)	Did not report outcome of interest
**276**	Ramos et al. (2021)	Did not report outcome of interest
**277**	Rani et al. (2024)	Did not meet the eligible population criteria
**278**	Rayzah et al. (2021)	Used improper outcome measure
**279**	Regestein et al. (2010)	Did not meet the eligible population criteria
**280**	Repa et al. (2018)	Full-text unavailable
**281**	Rezaei et al. (2018)	Did not report outcome of interest
**282**	Riaz et al. (2022)	Did not report outcome of interest
**283**	Ristovska et al. (2023)	Did not meet the eligible population criteria
**284**	Rosenberg et al. (2024)	Did not meet the eligible population criteria

**285**	Ruiter and Lichstein (2012)	Full-text unavailable
**286**	Ruivo Marques et al. (2019)	Did not report outcome of interest
**287**	Rutigliano et al. (2011)	Full-text unavailable
**288**	Sadigh et al. (2014)	Did not meet the eligible population criteria
**289**	C. Samaranayake et al. (2014)	Full-text unavailable
**290**	C. B. Samaranayake et al. (2014)	Did not report outcome of interest
**291**	Sánchez-Oviedo et al. (2021)	Did not report outcome of interest
**292**	Sasaki (2007)	Did not meet the eligible population criteria
**293**	Schepis et al. (2021)	Did not report outcome of interest
**294**	Schlarb and Grunwald (2018)	Full-text unavailable
**295**	Schlarb et al. (2017)	Did not report outcome of interest
**296**	Schmidt et al. (2010)	Did not meet the eligible population criteria
**297**	Schmidt et al. (2008)	Did not meet the eligible population criteria
**298**	Scotta et al. (2022)	Did not meet the eligible population criteria
**299**	Seehuus et al. (2024)	Did not report outcome of interest
**300**	Sehgal et al. (2016)	Had unrelated population
**301**	Serafin et al. (2021)	Did not meet the eligible population criteria
**302**	Sharma et al. (2021)	Did not meet the eligible population criteria
**303**	Sheaves et al. (2016)	Did not report outcome of interest
**304**	Shen et al. (2020)	Did not meet the eligible population criteria
**305**	Shen et al. (2021)	Did not meet the eligible population criteria
**306**	Shi et al. (2022)	Did not report outcome of interest
**307**	Shi et al. (2021)	Used improper outcome measure
**308**	Siddiqui et al. (2016)	Did not report outcome of interest
**309**	Sing and Wong (2010)	Used improper outcome measure
**310**	Sing and Wong (2011)	Did not report outcome of interest
**311**	Siomos et al. (2010)	Had unrelated population
**312**	Sivertsen et al. (2021)	Used improper outcome measure
**313**	Sivertsen et al. (2019)	Used improper outcome measure
**314**	Snyder et al. (2023)	Did not report outcome of interest
**315**	Solanki et al. (2023)	Did not meet the eligible population criteria
**316**	Sredniawa et al. (2019)	Did not meet the eligible population criteria
**317**	Steine et al. (2021)	Used improper outcome measure
**318**	Suganya et al. (2022)	Did not meet the eligible population criteria
**319**	Sun et al. (2024)	Did not report outcome of interest
**320**	Syafriani et al. (2015)	Full-text unavailable
**321**	Syed and Al-Rawi (2023)	Did not meet the eligible population criteria
**322**	Tadros et al. (2024)	Did not meet the eligible population criteria
**323**	Tafoya et al. (2013)	Did not report outcome of interest
**324**	Takuyo et al. (2023)	Had unrelated population
**325**	Tang et al. (2024)	Used improper outcome measure

**326**	Tang et al. (2022)	Did not meet the eligible population criteria
**327**	Taylor et al. (2013)	Used improper outcome measure
**328**	Taylor et al. (2011)	Used improper outcome measure
**329**	Thomas and Lichstein (2014)	Did not meet the eligible population criteria
**330**	Thomas (2015)	Did not meet the eligible population criteria
**331**	Tsai and Li (2004)	Did not report outcome of interest
**332**	Tsou and Chang (2019)	Had unrelated population
**333**	Turki et al. (2023)	Did not meet the eligible population criteria
**334**	Vedaa et al. (2019)	Used improper outcome measure
**335**	Veldi et al. (2005)	Did not report outcome of interest
**336**	Virnoche et al. (2024)	Full-text unavailable
**337**	Wang et al. (2022)	Used improper outcome measure
**338**	J. Wang et al. (2024)	Did not report outcome of interest
**339**	Wang et al. (2016)	Did not report outcome of interest
**340**	S. Wang et al. (2024)	Did not report outcome of interest
**341**	Wang and Sun (2024)	Did not meet the eligible population criteria
**342**	Williams et al. (2020)	Used improper outcome measure
**343**	Wu et al. (2022)	Did not meet the eligible population criteria
**344**	Xiao et al. (2021)	Did not meet the eligible population criteria
**345**	Xu et al. (2023)	Had unrelated population
**346**	Xu et al. (2021)	Did not meet the eligible population criteria
**347**	Yavuz et al. (2019)	Did not report outcome of interest
**348**	Yilmaz and Kugu (2022)	Did not meet the eligible population criteria
**349**	Younes et al. (2016)	Did not meet the eligible population criteria
**350**	Yu et al. (2020)	Did not meet the eligible population criteria
**351**	Yu et al. (2021)	Did not meet the eligible population criteria
**352**	Yuan et al. (2024)	Did not meet the eligible population criteria
**353**	Yun-Yi Yang and Jun (2018)	Did not meet the eligible population criteria
**354**	Yurasek et al. (2020)	Did not meet the eligible population criteria
**355**	Zafar et al. (2018)	Did not report outcome of interest
**356**	Zainab et al. (2020)	Did not meet the eligible population criteria
**357**	Zhai et al. (2018)	Did not report outcome of interest
**358**	D. Zhang et al. (2023)	Did not report outcome of interest
**359**	W. Zhang et al. (2023)	Used improper outcome measure
**360**	Zhang et al. (2024)	Did not meet the eligible population criteria
**361**	Zhang et al. (2021)	Did not meet the eligible population criteria
**362**	Zhao et al. (2022)	Did not meet the eligible population criteria
**363**	Zheng et al. (2021)	Did not meet the eligible population criteria
**364**	Zhou et al. (2019)	Full-text unavailable
**365**	Zou et al. (2023)	Did not meet the eligible population criteria
**366**	Zvolensky et al. (2021)	Did not report outcome of interest

Supplementary Table 8. Quality assessment summary table

Citations	Representativeness of the target population	Recruitment of participants	Adequate sample size	Description of subject and setting	Coverage of the identified sample	Objectivity of assessment	Reliability of the assessment	Appropriate statistical analysis	Adequate response rate/ management
Akram et al., 2023									
Al Maqbali et al., 2023									
Al Omari et al., 2022									
Alkhatatbeh et al., 2021									
Alqudah et al., 2019									
Alrashed et al., 2021									
Alrashed et al., 2022									
AlSamhori et al., 2024									
Alshammari et al., 2023									
Alshehri et al., 2024									
Ramón-Arbués et al., 2019									
Babicki et al., 2023									
Benham 2021									
Bodys-Cupak et al., 2022									
Chakraborty 2022									
Chen et al., 2023									

Dąbrowska-Galas et al., 2021									
Dongol et al., 2022									
Doss et al., 2020									
Durán-Agüero et al., 2019									
Eleftheriou et al., 2021									
Gaultney 2010									
Goweda et al., 2021									
Gress-Smith et al., 2015									
Gruba et al., 2021									
Hussein et al., 2024									
Iqbal et al., 2023									
Islam 2021									
Ito et al., 2022									
Kandeger et al., 2018									
Kim et al., 2022									
King et al., 2023									
Liao et al., 2022									
Liu et al., 2022									
Meneo et al., 2024									
Morales-Suárez-Varela et al., 2024									
Nadorff et al., 2011									
Parmar & Kumbhakar 2022									
Piro et al., 2018									
Ramón-Arbués et al., 2020									
Shakeel et al., 2019									

Solanki et al., 2023									
Sy et al., 2024									
Tanji & Kodama 2021									
Toubasi et al., 2023									
Vilca et al., 2022									
Xu & Jiang 2024									
Zhang et al., 2023									

References

Abdel Khalek, A., Al-Meshaan, O., & Al-Shatti, A. (1995). Themes of presleep thoughts among students of Kuwait University [Consciousness States 2380]. *Journal of the Social Sciences*, *23*(2), 63-112.

Abdel-Khalek, A. M. (2004). Prevalence of reported insomnia and its consequences in a survey of 5,044 adolescents in Kuwait. *Sleep*, *27*(4), 726-731. https://doi.org/10.1093/sleep/27.4.726

Abdel-Khalek, A. M. (2006). Prevalene of insomia compliants and its consequences in Kuwaiti college students. *Sleep and Hypnosis*, *8*(2).

Abdel-Khalek, A. M. (2008). The development and validation of the Arabic Scale of Insomnia (ASI). *Sleep and Hypnosis*, *10*(1), 3-10.

Abdulah, D. M., & Piro, R. S. (2018). Sleep disorders as primary and secondary factors in relation with daily functioning in medical students. *Annals of Saudi Medicine*, *38*(1), 57-64. https://doi.org/10.5144/0256-4947.2018.57

Ahmed, F. H. F., Shawky, A. E. S., & Al Sarraf, Z. K. (2022). Prevalence of Insomnia among university students in the United Arab Emirates. *Research Journal of Pharmacy and Technology*, *15*(8), 3401-3406. https://doi.org/10.52711/0974-360X.2022.00569

Ahmed, S., Ahsan, M. S., Khan, R., Hasan, M., Ferdous, F., Shahjahan, H., Hossain, M., Kar, A., & Hossain, K. (2021). Psychological impact of COVID-19 pandemic on frontline health care workers in Bangladesh: A cross-sectional study. *BJPsych Open*, *7*, S232-S233. https://doi.org/10.1192/bjo.2021.621

Ahrberg, K., Dresler, M., Niedermaier, S., Steiger, A., & Genzel, L. (2012). The interaction between sleep quality and academic performance. *Journal of Psychiatric Research*, *46*(12), 1618-1622. https://doi.org/10.1016/j.jpsychires.2012.09.008

Akram, U., Akram, A., Gardani, M., Ypsilanti, A., McCarty, K., Allen, S., & Lazuras, L. (2019). The relationship between depression and insomnia symptoms amongst a sample of UK university students. *Sleep Medicine Research*, *10*(1). https://doi.org/10.17241/smr.2019.00332

Al Salmani, A. A., Al Shidhani, A., Al Qassabi, S. S., Al Yaaribi, S. A., & Al Musharfi, A. M. (2020). Prevalence of sleep disorders among university students and its impact on academic performance. *International Journal of Adolescence and Youth 25*(1), 974-981. https://doi.org/10.1080/02673843.2020.1815550

Al-Mamun, F., Mamun, M. A., Hasan, M. E., Almerab, M. M., & Gozal, D. (2024). Exploring Sleep Duration and Insomnia Among Prospective University Students: A Study with Geographical Data and Machine Learning Techniques. *Nature and Science of Sleep 16*, 1235-1251. https://doi.org/10.2147/NSS.S481786

Alageel, A. A., Alyahya, R. A., Bahatheq, Y. A., Alzunaydi, N. A., Alghamdi, R. A., Alrahili, N. M., McIntyre, R. S., & Iacobucci, M. (2021). Smartphone addiction and associated factors among postgraduate students in an Arabic sample: A cross-sectional study. *BMC Psychiatry*, *21*. https://doi.org/10.1186/s12888-021-03285-0

Albasheer, O. B., Al Bahhawi, T., Ryani, M. A., Mohammed Arishi, A., Mohammed Hakami, O., Mohsen Maashi, S., Khairat Al-Khairat, H., Alganmy, O. M., Adnan Sahal, Y., Aaref Sharif, A., & Mahfouz, M. S. (2020). Prevalence of insomnia and relationship with depression, anxiety and stress among Jazan University

students: A cross-sectional study. *Cogent Psychology 7*(1). https://doi.org/10.1080/23311908.2020.1789424

Albikawi, Z. F. (2023). Fear Related to COVID-19, Mental Health Issues, and Predictors of Insomnia among Female Nursing College Students during the Pandemic. *Healthcar*, *11*(1), 174. https://doi.org/10.3390/healthcare11020174

Aldrin Joshua, A., Ganapathy, D., & Keerthi Sasanka, L. (2020). Insomnia among the dental college students. *International Journal of Pharmaceutical Research*, *12*(4), 2206 - 2215. https://doi.org/10.31838/ijpr/2020.12.04.309

Alfadeel, M. A., Alqahtani, N., Alhudaib, M., Almudhee, S., Alghamdi, A., Jadou, N., Aljarbou, R., bin Sabit, D., Alrajih, H., & Alshammari, S. (2019). The Prevalence of Insomnia Among Female Medical Students of Almaarefa Colleges in Riyadh City – Kingdom of Saudi Arabia (2015–2016). *Indo American Journal of Pharmaceutical Sciences 6*(2), 3377-3391. https://doi.org/10.5281/zenodo.2560901

Alfawaz, H. A., Wani, K., Aljumah, A. A., Aldisi, D., Ansari, M. G. A., Yakout, S. M., Sabico, S., & Al-Daghri, N. M. (2021). Psychological well-being during COVID-19 lockdown: Insights from a Saudi State University's Academic Community. *Journal of King Saud University Science 33*(1), Article 101262. https://doi.org/10.1016/j.jksus.2020.101262

AlHadi, A. N., & Alhuwaydi, A. M. (2022). Insomnia Prevalence and Associated Factors Among University Students in Saudi Arabia During the COVID-19 Pandemic and Lockdown: A Large-Scale Survey. *Nature and Science of Sleep 14*, 1651-1663. https://doi.org/doi:10.2147/NSS.S380972

Alkahtani, R. F., Alomar, A. A., Alkanhal, A. F., Alhinti, M. F., Alatoui, S. E., Alrashidi, R. R., & Saleh, A. (2022). Prevalence of Anxiety, Depression, and Sleep Disturbances Associated With the COVID-19 Outbreak in Riyadh, Saudi Arabia. *Cureus*, *14*(5), e24838. https://doi.org/10.7759/cureus.24838

Almansour, A., AlJammaz, F., Ahmeda, A., Alfawaz, M., Abdulsalam, K., AlSheikh, A., Aljaloud, M., & Alkhudhayr, M. (2020). The Prevalence of Sleep Deprivation and its influence on Students' Life Attending Medical School at King Saud University. *International Journal of Pharmaceutical and Phytopharmacological Research 10*(5), 149-156.

Almusa, H. A. (2021). Insomnia and social network use among secondary school female students in Abha Sector. *World Family Medicine*, *19*(2), 102-111. https://doi.org/10.5742/MEWFM.2021.93989

Alomri, R. M., & Alghamdi, Y. (2024). The Prevalence and Predictors of Sleep Disorders and Their Impact on Academic Performance Among Saudi University Students: A Cross-Sectional Study. *The Cureus Journal of Medical Science*, *16*(5), Article e61334. https://doi.org/10.7759/cureus.61334

Alqahtani, J. S., AlRabeeah, S. M., Aldhahir, A. M., Siraj, R., Aldabayan, Y. S., Alghamdi, S. M., Alqahtani, A. S., Alsaif, S. S., Naser, A. Y., & Alwafi, H. (2022). Sleep Quality, Insomnia, Anxiety, Fatigue, Stress, Memory and Active Coping during the COVID-19 Pandemic. *International Journal of Environmental Research and Public Health*, *19*(9), Article 4940. https://doi.org/10.3390/ijerph19094940

Alsaadi, S. M. (2022). Musculoskeletal Pain in Undergraduate Students Is Significantly Associated with Psychological Distress and Poor Sleep Quality. *International Journal of Environmental Research and Public Health*, *19*(21). https://doi.org/10.3390/ijerph192113929

Alshammari, T. K., Rogowska, A. M., Alobaid, A. M., Alharthi, N. W., Albaker, A. B., & Alshammari, M. A. (2024). Examining Anxiety and Insomnia in Internship Students and Their Association with Internet Gaming Disorder. *Journal of Clinical Medicine*, *13*(14), 4054. https://doi.org/10.3390/jcm13144054

Alshammari, T. K., Rogowska, A. M., Basharahil, R. F., Alomar, S. F., Alseraye, S. S., Al Juffali, L. A., Alrasheed, N. M., & Alshammari, M. A. (2023). Examining bedtime procrastination, study engagement, and studyholism in undergraduate students, and their association with insomnia. *Frontiers in Psychology*, *13*, Article 1111038. https://doi.org/10.3389/fpsyg.2022.1111038

Alsuhaymi, Z. S., Alreheli, A. Q., Alawfi, A. M., Alhazmi, A. M., Aljuhani, R. Z., Alruhaili, E. M. S., & Alsuhimi, S. S. (2019). Impact of sleep habits on academic achievement among fourth-year medical students at Taibah University. *Indo American Journal of Pharmaceutical Sciences*, *6*(1), 790-800. https://doi.org/10.5281/zenodo.2537603

Alyoubi, A., Halstead, E. J., Zambelli, Z., & Dimitriou, D. (2021). The impact of the covid-19 pandemic on students' mental health and sleep in Saudi Arabia. *International Journal of Environmental Research and Public Health*, *18*(17), 9344. https://doi.org/10.3390/ijerph18179344

Amaral, A. P., Soares, M. J., Pinto, A. M., Pereira, A. T., Madeira, N., Bos, S. C., Marques, M., Roque, C., & Macedo, A. (2018). Sleep difficulties in college students: The role of stress, affect and cognitive processes. *Psychiatry Research*, *260*, 331-337. https://doi.org/10.1016/j.psychres.2017.11.072

Amelia, R., Harahap, J., & Wijaya, H. (2023). The relationship between sleep hygiene and the prevalence of insomnia in medical students during the COVID-19 pandemic at the Faculty of Medicine, Universitas Sumatera Utara, Medan, Indonesia. *Family Medicine and Primary Care Review*, *25*(1), 14-17. https://doi.org/10.5114/fmpcr.2023.125486

Amlak, B. T., Bitew, M. S., Getnet, A., Yitayew, F. M., Terefe, T. F., Tarekegn, T. T., Mihret, A. G., Geleta, O. T., Alemu, G. G., GebreEyesus, F. A., & Tsegaye, D. (2022). The magnitude of mental distress and associated factors among a school of medicine and college of health sciences students at Debre Markos University, 2021. *PLoS One*, *17*(9), Article e0275120. https://doi.org/10.1371/journal.pone.0275120

Andorko, N. D., Mittal, V., Thompson, E., Denenny, D., Epstein, G., Demro, C., Wilson, C., Sun, S. Y., Klingaman, E. A., DeVylder, J., Oh, H., Postolache, T. T., Reeves, G. M., & Schiffman, J. (2017). The association between sleep dysfunction and psychosis-like experiences among college students. *Psychiatry Research*, *248*, 6-12. https://doi.org/10.1016/j.psychres.2016.12.009

Angelone, A. M., Mattei, A., Sbarbati, M., & Di Orio, F. (2011). Prevalence and correlates for self-reported sleep problems among nursing students. *Journal of Preventive Medicine and Hygiene*, *52*(4), 201 - 208.

Anupama, M., Kulkarni, H., Nisarga, V., & Sushravya. (2022). Perceived Stress and Insomnia among Medical Residents in South India: A Cross-Sectional Study. *Annals of Indian Psychiatry*, *6*(2), 142-148. https://doi.org/10.4103/aip.aip_140_21

Anwer, S., Li, H., Antwi-Afari, M. F., Abu Shaphe, M., Alghadir, A., & Wong, A. Y. L. (2021). Evaluation of Sleep Habits, Generalized Anxiety, Perceived Stress, and Research Outputs Among Postgraduate Research Students in Hong Kong During the Coronavirus (COVID-19) Pandemic. *Journal of Multidisciplinary*

Healthcare, *14*, 3135-3149. https://doi.org/10.2147/JMDH.S325479

Apaza-Panca, C. M., Maquera-Luque, P. J., Huanca-Frías, J. O., Supo-Quispe, L. A., Tavara-Ramos, A. P., Dextre-Martínez, W. R., & Saldaña-Acosta, O. A. (2021). Comparison of psychosocial factors in university students from Loreto, Ancash, Moquegua and Puno confinement by Covid-19, Peru. *Cuestiones Politicas*, *39*(68), 51-69. https://doi.org/10.46398/cuestpol.3968.02

Araujo, M. F., Freitas, R. W., Lima, A. C., Pereira, D. C., Zanetti, M. L., & Damasceno, M. M. (2014). Health indicators associated with poor sleep quality among university students. *Revista da Escola de Enfermagem da U S P*, *48*(6). https://doi.org/10.1590/S0080-623420140000700017

Arbinaga, F., Fernández-Cuenca, S., Fernández-Ozcorta, E. J., Toscano-Hermoso, M. D., & Joaquin-Mingorance, M. (2019). Level of physical activity and sleep characteristics in university students. *Sleep Science*, *12*(4), 265-271. https://doi.org/10.5935/1984-0063.20190092

Assefa, Z. M., Haile, T. G., Wazema, D. H., Tafese, W. T., Berrie, F. W., Beketie, E. D., Hailemariam, B. Z., Zewudie, B. T., Teke, N. E., & Metebo, K. N. (2021). Mental Health Disorders During COVID-19 Pandemic Among Southwest Ethiopia University Students: An Institutional-Based Cross-Sectional Study. *Sage Open Nursing*, *7*. https://doi.org/10.1177/23779608211064374

Atlam, S. A., & Elsabagh, H. M. (2020). Sleep habits and their association with daytime sleepiness among medical students of Tanta University, Egypt. *Epidemiologic Methods*, *9*(1), 20190034. https://doi.org/10.1515/em-2019-0034

Baalaraj, F. S., Bin Laswad, B. M., Habeeb, D. E., Alshaikh, M. G., Almatrafi, M. H., Alafifi, M. M., & Shatla, M. M. (2022). Prevalence and different management of sleep disorders among medical students, Umm Alqura University Makkah, Saudi Arabia. *Medical Science*, *26*(128). https://doi.org/doi:10.54905/disssi/v26i128/ms422e2424

Babicki, M., Piotrowski, P., & Mastalerz-Migas, A. (2022). Assessment of Insomnia Symptoms, Quality of Life, Daytime Sleepiness, and Psychoactive Substance Use among Polish Students: A Cross-Sectional Online Survey for Years 2016-2021 before and during COVID-19 Pandemic. *Journal of Clinical Medicine*, *11*(8), 2106. https://doi.org/10.3390/jcm11082106

Backovic, D. V., Maksimovic, M., Davidovic, D., Zivojinovic, J. I., & Stevanovic, D. (2013). Stress and Mental Health among Medical Students. *Srpski Arhiv Za Celokupno Lekarstvo*, *141*(11-12), 780-784. https://doi.org/10.2298/SARH1312780B

Badellino, H., Gobbo, M. E., Torres, E., Aschieri, M. E., Biotti, M., Alvarez, V., Gigante, C., & Cachiarelli, M. (2024). Psychological and social effects on long term quarantined college students: Prevalence, correlated factors and coping skills. *Revista Colombiana de Psiquiatria*. https://doi.org/10.1016/j.rcp.2023.10.004

Baker, L. D., Baldwin, D. S., & Garner, M. (2015). Daytime intrusive thoughts and subjective insomnia symptoms. *Psychiatry Research*, *229*(3), 1038-1042. https://doi.org/10.1016/j.psychres.2015.02.022

Bastien, C. H., Ellis, J. G., Athey, A., Chakravorty, S., Robbins, R., Knowlden, A. P., Charest, J., & Grandner, M. A. (2019). Driving after drinking alcohol associated with insufficient sleep and insomnia among student athletes and non-athletes. *Brain Sciences*, *9*(2), 46. https://doi.org/10.3390/brainsci9020046

https://doi.org/10.3390/brainsci9020046

Baylor, A., Thompson, E., Griffin, S., & Rybarczyk, B. (2016). Insomnia and behaviorally induced insufficient sleep's relationship to mood disorders in college students. *Sleep*, *39*(SUPPL. 1), A298. (30th Annual Meeting of the Associated Professional Sleep Societies, LLC, SLEEP 2016. Denver, CO United States.)

Becker, S. P., Dvorsky, M. R., Holdaway, A. S., & Luebbe, A. M. (2018). Sleep problems and suicidal behaviors in college students. *Journal of Psychiatric Research*, *99*, 122-128. https://doi.org/10.1016/j.jpsychires.2018.01.009

Belingheri, M., Luciani, M., Ausili, D., Paladino, M. E., Di Mauro, S., De Vito, G., & Riva, M. A. (2022). Sleep disorders and night-shift work in nursing students: a cross-sectional study. *Medicina del Lavoro*, *113*(1), Article e2022003. https://doi.org/10.23749/mdl.v113i1.12150

Belingheri, M., Pellegrini, A., Facchetti, R., De Vito, G., Cesana, G., & Riva, M. A. (2020). Self-reported prevalence of sleep disorders among medical and nursing students [Physical & Somatic Disorders 3290
Health & Mental Health Personnel Issues 3400]. *Occupational Medicine*, *70*(2), 127-130. https://doi.org/10.1093/occmed/kqaa011

Benham, G. (2021). Stress and sleep in college students prior to and during the COVID-19 pandemic. *Stress and Health : Journal of the International Society for the Investigation of Stress*, *37*(3), 504-515. https://doi.org/10.1002/smi.3016

Benham, G. (2022). Sleep paralysis in college students. *Journal of American College Health*, *70*(5), 1286-1291. https://doi.org/10.1080/07448481.2020.1799807

Benham, G., Barajas, C., Martinez, G. E., & Gonzalez, M. (2019). Sleep paralysis in college students: Prevalence, subjective experiences, and correlates. *Sleep*, *42*(Supplement 1). https://doi.org/10.1093/sleep/zsz067.646

Bhakat, P., & Das, K. (2023). Status of mental health among college and university students during first and second wave of COVID-19 outbreak in India: A cross-sectional study. *Journal of Affective Disorders Reports*, *12*, 100494. https://doi.org/10.1016/j.jadr.2023.100494

Brown, F. C., Buboltz, W. C., Jr., & Soper, B. (2002). Relationship of sleep hygiene awareness, sleep hygiene practices, and sleep quality in university students. *Behavioral Medicine*, *28*(1), 33-33. https://doi.org/10.1080/08964280209596396

Brunk, D. (2019). Insomnia common among transgender college students. *Chest Physician*, *14*(7), 18-18.

Buboltz, W. C., Jr., Brown, F., & Soper, B. (2001). Sleep habits and patterns of college students: A preliminary study [Physical & Somatic Disorders 3290 Classroom Dynamics & Student Adjustment & Attitudes 3560]. *Journal of American College Health*, *50*(3), 131-135. https://doi.org/10.1080/07448480109596017

Buckner, J. D., Bernert, R. A., Cromer, K. R., Joiner, T. E., & Schmidt, N. B. (2008). Social anxiety and insomnia: The mediating role of depressive symptoms [Psychological & Physical Disorders 3200]. *Depression and Anxiety*, *25*(2), 124-130. https://doi.org/10.1002/da.20282

Calderon, A., Baik, S. Y., Ng, M. H. S., Fitzsimmons-Craft, E. E., Eisenberg, D., Wilfley, D. E., Taylor, C. B., & Newman, M. G. (2024). Machine learning and Bayesian network analyses identifies associations with

insomnia in a national sample of 31,285 treatment-seeking college students. *BMC Psychiatry*, *24*(1), 656. https://doi.org/10.1186/s12888-024-06074-7

Carney, C. E., Edinger, J. D., Meyer, B., Lindman, L., & Istre, T. (2006). Daily activities and sleep quality in college students. *Chronobiology International*, *23*(3), 623-637. https://doi.org/10.1080/07420520600650695

Carpi, M., Cianfarani, C., & Vestri, A. (2022). Sleep Quality and Its Associations with Physical and Mental Health-Related Quality of Life among University Students: A Cross-Sectional Study. *International Journal of Environmental Research and Public Health*, *19*(5), 2874. https://doi.org/10.3390/ijerph19052874

Carpi, M., Marques, D. R., Milanese, A., & Vestri, A. (2022). Sleep Quality and Insomnia Severity among Italian University Students: A Latent Profile Analysis. *Journal of Clinical Medicine*, *11*(14). https://doi.org/doi:10.3390/jcm11144069

Carrillo Ibarra, J., Valdez Talavera, L. A., Vazquez Urbano, H., Franco Jimenez, J., De La Pena Sifuentes, A., & Martinez Coronado, J. (2010). Depression, suicidal ideation and insomnia in Saltillo university, important public health problems. *Revista Mexicana de Neurociencia*, *11*(1), 30-32.

Carrión-Pantoja, S., Prados, G., Chouchou, F., Holguín, M., Mendoza-Vinces, Á., Expósito-Ruiz, M., & Fernández-Puerta, L. (2022). Insomnia Symptoms, Sleep Hygiene, Mental Health, and Academic Performance in Spanish University Students: A Cross-Sectional Study. *Journal of Clinical Medicine*, *11*(7). https://doi.org/10.3390/jcm11071989

Castaldelli-Maia, J. M., Lewis, T., Marques dos Santos, N., Picon, F., Kadhum, M., Farrell, S. M., Molodynski, A., & Ventriglio, A. (2019). Stressors, psychological distress, and mental health problems amongst Brazilian medical students. *International Review of Psychiatry*, *31*(7-8). https://doi.org/10.1080/09540261.2019.1669335

Castelnuovo, A., Mombelli, S., Bottoni, D., Somma, A., Fossati, A., Ferini-Strambi, L., & Marelli, S. (2021). Quality of sleep is the only predictor of suicide during COVID-19 lockdown in university students? *Sleep*, *44*(SUPPL 2), A272. https://doi.org/10.1093/sleep/zsab072.694

Cebrino, J., & Portero de la Cruz, S. (2023). Psychological impact of COVID-19 and determinants among Spanish university students. *Frontiers in Public Health*, *11*, 1252849. https://doi.org/10.3389/fpubh.2023.1252849

Celik, N., Ceylan, B., Unsal, A., & Cagan, O. (2019). Depression in health college students: Relationship factors and sleep quality [Affective Disorders 3211]. *Psychology, Health & Medicine*, *24*(5), 625-630. https://doi.org/10.1080/13548506.2018.1546881

Chan-Chee, C., Bayon, V., Bloch, J., Beck, F., Giordanella, J. P., & Leger, D. (2011). Epidemiology of insomnia in France. *Revue D Epidemiologie Et De Sante Publique*, *59*(6), 409-422. https://doi.org/10.1016/j.respe.2011.05.005

Chandler, L., McEnery, K. A. M., Toro, C., Singh, S. P., Meyer, C., & Tang, N. K. Y. (2023). Do mental health symptoms during the pandemic predict university non-completion in a sample of UK students? A prospective study [Classroom Dynamics & Student Adjustment & Attitudes 3560]. *Education Sciences*, *13*(12), 1-15. https://doi.org/10.3390/educsci13121227

Chen, C., He, Z., Xu, B., Shao, J., & Wang, D. (2023). A latent profile analysis of sleep disturbance in relation to

mental health among college students in China. *Frontiers in Public Health*, *11*, 1107692. https://doi.org/10.3389/fpubh.2023.1107692

Chen, M., Zhang, X., Liu, X., Chen, Y., Liu, R., Peng, L., & Li, M. (2024). The association between insomnia symptoms and cognitive flexibility among undergraduates: An event-related potential study. *Sleep Medicine*, *121*, 343-351. https://doi.org/10.1016/j.sleep.2024.07.013

Chen, S., Liao, J. Q., Ran, F., Wang, X., Liu, Y. L., & Zhang, W. (2024). Longitudinal Associations between Future Time Perspective, Sleep Problems, and Depressive Symptoms among Chinese College Students: Between- and within-Person Effects. *Journal of Youth and Adolescence*. https://doi.org/10.1007/s10964-024-02078-4

Cheng, J. Z., Liao, M., He, Z. P., Xiong, R., Ju, Y. M., Liu, J., Liu, B. S., Wu, B., & Zhang, Y. (2023). Mental health and cognitive function among medical students after the COVID-19 pandemic in China. *Frontiers in Public Health*, *11*, Article 1233975. https://doi.org/10.3389/fpubh.2023.1233975

Cheng, S. H., Shih, C.-C., Lee, I. H., Hou, Y.-W., Chen, K. C., Chen, K.-T., Yang, Y. K., & Yang, Y. C. (2012). A study on the sleep quality of incoming university students. *Psychiatry Research*, *197*(3), 270-274. https://doi.org/10.1016/j.psychres.2011.08.011

Cheraghi, F., & Shamsaei, F. (2008). Prevalence of insomnia of nursing and midwifery students. *International Journal of Psychology*, *43*(3-4), 267-267.

Choueiry, N., Salamoun, T., Jabbour, H., El Osta, N., Hajj, A., & Khabbaz, L. R. (2016). Insomnia and relationship with anxiety in university students: A cross-sectional designed study. *PLoS One*, *11*(2). https://doi.org/10.1371/journal.pone.0149643

Chowdhury, A. H., Rad, D., & Rahman, M. S. (2024). Predicting anxiety, depression, and insomnia among Bangladeshi university students using tree-based machine learning models. *Health Science Reports*, *7*(4). https://doi.org/doi:10.1002/hsr2.2037

Christodoulou, N., Maruani, J., d'Ortho, M. P., Lejoyeux, M., & Geoffroy, P. A. (2023). Sleep quality of medical students and relationships with academic performances. *Encephale-Revue De Psychiatrie Clinique Biologique Et Therapeutique*, *49*(1), 9-14. https://doi.org/10.1016/j.encep.2021.09.006

Chu, C., Hom, M. A., Rogers, M. L., Ringer, F. B., Hames, J. L., Suh, S., & Joiner, T. E. (2016). Is insomnia lonely? Exploring thwarted belongingness as an explanatory link between insomnia and suicidal ideation in a sample of South Korean university students. *Journal of Clinical Sleep Medicine*, *12*(5). https://doi.org/10.5664/jcsm.5784

Coelho, J., Montagni, I., Micoulaud-Franchi, J. A., Plancoulaine, S., & Tzourio, C. (2023). Study of the association between cannabis use and sleep disturbances in a large sample of University students. *Psychiatry Research*, *322*, 115096. https://doi.org/10.1016/j.psychres.2023.115096

Copaja-Corzo, C., Miranda-Chavez, B., Vizcarra-Jiménez, D., Hueda-Zavaleta, M., Rivarola-Hidalgo, M., Parihuana-Travezaño, E. G., & Taype-Rondan, A. (2022). Sleep Disorders and Their Associated Factors during the COVID-19 Pandemic: Data from Peruvian Medical Students. *Medicina-Lithuania*, *58*(10), Article 1325. https://doi.org/10.3390/medicina58101325

Correa Rangel, T., Falcao Raposo, M. C., & Sampaio Rocha-Filho, P. A. (2021). The prevalence and severity of

insomnia in university students and their associations with migraine, tension-type headache, anxiety and depression disorders: A cross-sectional study. *Sleep Medicine*, *88*, 241-246. https://doi.org/10.1016/j.sleep.2021.10.029

Correa Rangel, T., Falcao Raposo, M. C., & Sampaio Rocha-Filho, P. A. (2022). Internet addiction, headache, and insomnia in university students: a cross-sectional study. *Neurological Sciences*, *43*(2). https://doi.org/10.1007/s10072-021-05377-x

da Silva Cardoso, G. M., da Silva, M. P. F. N., de Castro Correa, C., & Weber, S. A. T. (2022). Insomnia and excessive daytime sleepiness in medical students: consequences of the use of technologies? *Sleep Science*, *15*. https://doi.org/10.5935/1984-0063.20220008

da Silva-Fonseca, V. A., Vasquez, F. B., Seixas, A., Jean-Louis, G., da Silva-Fonseca, M. S., Sladek, L., da Rocha, E. M. S., Santos, R. M. M., & de Aguiar, A. S. (2021). Binge drinking and insomnia in students from health sciences at one university in rio de janeiro, brazil. *Brazilian Journal of Medical and Biological Research*, *54*(8), e10679. https://doi.org/10.1590/1414-431X202010679

Daghigh, A. (2024). Beyond sleepless nights: Unraveling the complexity of alexithymia and suicide risk among university students. *Brain and Behavior*, *14*(4), e3476. https://doi.org/10.1002/brb3.3476

Dagnew, B., Andualem, Z., & Dagne, H. (2020). Excessive daytime sleepiness and its predictors among medical and health science students of University of Gondar, Northwest Ethiopia: institution-based cross-sectional study. *Health & Quality of Life Outcomes*, *18*(1). https://doi.org/10.1186/s12955-020-01553-3

De Souza, J. C. R. P. (1996). Quality of life and insomnia in university psychology students. *Human Psychopharmacology*, *11*(3), 169-184. https://doi.org/10.1002/%28SICI%291099-1077%28199605%2911:3%3C169::AID-HUP785%3E3.0.CO;2-F

Deng, A. P., Wang, C., Cai, J., Deng, Z. Y., Mu, Y. F., Song, H. J., Meng, Y. J., Meng, X. D., Huang, X. H., Zhang, L., Huang, Y., Zhang, W., Chen, J., & Ran, M. S. (2023). Effects of internet addiction and academic satisfaction on mental health among college students after the lifting of COVID-19 restrictions in China. *Frontiers in Psychiatry*, *14*, 1243619. https://doi.org/10.3389/fpsyt.2023.1243619

Denis, D., Poerio, G. L., Derveeuw, S., Badini, I., & Gregory, A. M. (2019). Associations between exploding head syndrome and measures of sleep quality and experiences, dissociation, and well-being [Physical & Somatic Disorders 3290]. *Sleep: Journal of Sleep and Sleep Disorders Research*, *42*(2), 1-11. https://doi.org/10.1093/sleep/zsy216

Dewan, H., Abdul, N. S., Mandal, N. B., Nasar, A., Mogla, S., Loganathan, J., & Dutta, P. (2022). Sleep disorders among dental students: An original research. *Journal of Pharmacy and Bioallied Sciences*, *14*, 275-276. https://doi.org/10.4103/jpbs.jpbs_729_21

Doos Ali Vand, H., Gharraee, B., Farid, A. A., & Bandi, M. G. (2014). Prediction of insomnia severity based on cognitive, metacognitive, and emotional variables in college students. *Explore (NY)*, *10*(4), 233-240. https://doi.org/10.1016/j.explore.2014.04.005

Duan, H., Gong, M., Zhang, Q., Huang, X., & Wan, B. (2022). Research on sleep status, body mass index, anxiety and depression of college students during the post-pandemic era in Wuhan, China. *Journal of Affective*

Disorders, *301*, 189-192. https://doi.org/10.1016/j.jad.2022.01.015

El Ansari, W., Khalil, K., & Stock, C. (2014). Symptoms and health complaints and their association with perceived stressors among students at nine libyan universities. *International Journal of Environmental Research and Public Health*, *11*(12). https://doi.org/10.3390/ijerph111212088

El Hangouche, A. J., Jniene, A., Aboudrar, S., Errguig, L., Rkain, H., Cherti, M., & Dakka, T. (2018). Relationship between poor quality sleep, excessive daytime sleepiness and low academic performance in medical students. *Advances In Medical Education And Practice*, *9*, 631-638. https://doi.org/10.2147/AMEP.S162350

El Sahly, R., Ahmed, A., Amer, S., & Alsaeiti, K. (2020). Assessment of insomnia and sleep quality among medical students-benghazi university: A cross-sectional study. *Apollo Medicine*, *17*(2), 73-77. https://doi.org/10.4103/am.am_22_20

El-Anzi, F. O. (2006). Insomnia in Relation to Depression and Somatic Symptoms [Psychological & Physical Disorders 3200]. *Psychological Reports*, *99*(1), 171-175. https://doi.org/10.2466/PR0.99.5.171-175

Eleftheriou, A., Rokou, A., Arvaniti, A., Nena, E., & Steiropoulos, P. (2021). Sleep Quality and Mental Health of Medical Students in Greece During the COVID-19 Pandemic. *Frontiers in Public Health*, *9*, Article 775374. https://doi.org/10.3389/fpubh.2021.775374

Ergin, N., Kiliç, B. B., Ergin, A., & Varli, S. (2022). Sleep quality and related factors including restless leg syndrome in medical students and residents in a Turkish university. *Sleep and Breathing*, *26*(3), 1299-1307. https://doi.org/10.1007/s11325-021-02437-6

Etindele-Sosso, F. A. (2020). Insomnia, excessive daytime sleepiness, anxiety, depression andsocioeconomicstatusamongcustomerserviceemployees in Canada. *Sleep Science*, *13*(1). https://doi.org/10.5935/1984-0063.20190133

Eva, F. N., Dev, D., Roy, S., Tipu, S. K., Medha, M. R. B., Poonya, P. T. H., Juthi, I. J., Nowrin, J. H., Eaasvar, J. C., Sumat, T., Dey, D. M., Chowdhury, S., Iktidar, M. A., & Hawlader, M. D. H. (2024). Prevalence and predictors of functional gastrointestinal disorder among the undergraduate students of Bangladesh. *PLoS One*, *19*(12), e0315687. https://doi.org/10.1371/journal.pone.0315687

Falavigna, A., De Souza Bezerra, M. L., Teles, A. R., Kleber, F. D., Velho, M. C., Steiner, B., Beckenkamp, N. L., Lazzaretti, L., Barazzetti, D., Abruzzi, F., Baseggio, N., De Braga, G. L., Ferrari, P., & De Souza, M. V. (2011). Sleep disorders among undergraduate students in Southern Brazil. *Sleep and Breathing*, *15*(3). https://doi.org/10.1007/s11325-010-0396-6

Faria, C., Ferreira, M., Allen, A., & Azevedo, M. H. (2014). Factors associated with self reported insomnia and sleep loss over worry in young adults. *Journal of Sleep Research*, *23*, 131. https://doi.org/10.1111/jsr.12213

Feher, A., Fejes, E., Kapus, K., Jancsak, C., Nagy, G. D., Horvath, L., Tibold, A., & Feher, G. (2023). The association of problematic usage of the internet with burnout, depression, insomnia, and quality of life among Hungarian high school students. *Frontiers in Public Health*, *11*, Article 1167308. https://doi.org/10.3389/fpubh.2023.1167308

Fernandes, A. C. A., Padilha, D. D. M. M., de Moura, A. C. M. A., de Aquino, C. E. F., de Araujo Lima, I. B., &

Mota-Rolim, S. A. (2022). COVID-19 pandemic decreased sleep quality of medical students. *Sleep Science*, *15*(4). https://doi.org/10.5935/1984-0063.20220075

Fernandez-Mendoza, J., Vela-Bueno, A., Vgontzas, A. N., Olavarrieta-Bernardino, S., Ramos-Platon, M. J., Bixler, E. O., & De la Cruz-Troca, J. J. (2009). Nighttime sleep and daytime functioning correlates of the insomnia complaint in young adults. *Journal of Adolescence*, *32*(5), 1059-1074. https://doi.org/10.1016/j.adolescence.2009.03.005

Fila-Witecka, K., Senczyszyn, A., Kolodziejczyk, A., Ciulkowicz, M., Maciaszek, J., Misiak, B., Szczesniak, D., & Rymaszewska, J. (2021). Lifestyle changes among polish university students during the covid-19 pandemic. *International Journal of Environmental Research and Public Health*, *18*(18), 9571. https://doi.org/10.3390/ijerph18189571

Floyd, V., & Vargas, I. (2024). Evaluating a mobile application based intervention for insomnia in college students: a preliminary study. *Journal of American College Health*. https://doi.org/10.1080/07448481.2024.2423225

Foster, H., Stevenson, J., & Akram, U. (2023). Prevalence and Psychiatric Correlates of Illicit Substance Use in UK Undergraduate Students. *Brain Sciences*, *13*(2), 360. https://doi.org/10.3390/brainsci13020360

Friedrich, A., Classen, M., & Schlarb, A. A. (2016). Tell me what you study, and I'll tell you how you sleep: On the relationship between field of study and sleep. *Somnologie*, *20*(4). https://doi.org/10.1007/s11818-016-0076-2

Gao, R., Wang, H., Liu, S., Wang, X., Song, S., & Wang, Y. (2024). Study on anxiety, depression, and sleep conditions and their interrelations among vocational college students during the COVID-19 pandemic management normalization. *Frontiers in Public Health*, *12*, 1385639. https://doi.org/10.3389/fpubh.2024.1385639

Gao, R., Wang, H., Liu, S., Wang, X., Xiong, X., Song, S. Y., & Wang, Y. (2024). Mental well-being and sleep quality among vocational college students in Sichuan, China during standardized COVID-19 management measures. *Frontiers in Public Health*, *12*, 1387247. https://doi.org/10.3389/fpubh.2024.1387247

Gaultney, J. F. (2010). The prevalence of sleep disorders in college students: impact on academic performance. *Journal of American College Health*, *59*(2), 91-97. https://doi.org/10.1080/07448481.2010.483708

Gaultney, J. F., Gray, D., & Daley, K. (2012). Refining a survey measure of risk for narcolepsy. *Sleep*, *35*(SUPPL. 1), A130. (26th Annual Meeting of the Associated Professional Sleep Societies, LLC, SLEEP 2012. Boston, MA United States.)

Gellis, L. A. (2011). Nighttime cognitive distraction and insomnia severity among university students. *Sleep*, *34*(SUPPL. 1), A184.

Gellis, L. A., Park, A., Stotsky, M. T., & Taylor, D. J. (2014). Associations between sleep hygiene and insomnia severity in college students: Cross-sectional and prospective analyses [Group & Interpersonal Processes 3020 Physical & Somatic Disorders 3290]. *Behavior Therapy*, *45*(6), 806-816. https://doi.org/10.1016/j.beth.2014.05.002

Gemnani, V. K., Ambwani, J. K., Shaikh, B. A., Larik, S. A., Shaikh, M. A., & Malik, E. (2019). Impact of insomnia on academic performance among undergraduate medical students of Chandka Medical College

(Shaheed Mohtarma Benazir Bhutto Medical University) Larkana. *Medical Forum Monthly*, *30*(1).

Ghrouz, A. K., Noohu, M. M., Manzar, M. D., Bekele, B. B., Pandi-Perumal, S. R., & Bahammam, A. S. (2021). Short-term insomnia symptoms are associated with level and not type of physical activity in a sample of Indian college students. *Journal of Preventive Medicine and Hygiene*, *62*(2), E447-E454. https://doi.org/10.15167/2421-4248/jpmh2021.62.2.1893

Ghrouz, A. K., Noohu, M. M., Manzar, M. D., Spence, D. W., BaHammam, A. S., & Pandi-Perumal, S. R. (2019). Physical activity and sleep quality in relation to mental health among college students. *Sleep and Breathing*, *23*(2), 627-634. https://doi.org/10.1007/s11325-019-01780-z

Gianfredi, V., Nucci, D., Tonzani, A., Amodeo, R., Benvenuti, A. L., Villarini, M., & Moretti, M. (2018). Sleep disorder, Mediterranean Diet and learning performance among nursing students: inSOMNIA, a cross-sectional study. *Annali Di Igiene Medicina Preventiva E Di Comunita*, *30*(6), 470-481. https://doi.org/10.7416/ai.2018.2258

Gilstrap, S. R., Hobson, J. M., Dark, H. E., Gloston, G. F., Cody, S. L., Goodin, B. R., & Thomas, S. J. (2023). Disordered sleep and its association with academic performance and functioning [Academic Learning & Achievement 3550]. *Sleep and Biological Rhythms*, *21*(1), 113-123. https://doi.org/10.1007/s41105-022-00423-3

Goel, A., Moinuddin, A., Tiwari, R., Sethi, Y., Suhail, M. K., Mohan, A., Kaka, N., Sarthi, P., Dutt, R., Ahmad, S. F., Attia, S. M., Emran, T. B., Chopra, H., & Greig, N. H. (2023). Effect of Smartphone Use on Sleep in Undergraduate Medical Students: A Cross-Sectional Study. *Healthcare*, *11*(21), Article 2891. https://doi.org/10.3390/healthcare11212891

Goel, N. J., Sadeh-Sharvit, S., Trockel, M., Flatt, R. E., Fitzsimmons-Craft, E. E., Balantekin, K. N., Monterubio, G. E., Firebaugh, M.-L., Wilfley, D. E., & Taylor, C. B. (2021). Depression and anxiety mediate the relationship between insomnia and eating disorders in college women. *Journal of American College Health*, *69*(8), 976-981. https://doi.org/10.1080/07448481.2019.1710152

Gonçalves, A. M., Cabral, L. R., Ferreira, M. C., Coutinho, E., & Duarte, J. C. (2017). *Influence Of Internet Use On The Quality Of Sleep Of University Students* Inted2017: 11th International Technology, Education And Development Conference.

Goodhines, P. A., Gellis, L. A., Kim, J., Fucito, L. M., & Park, A. (2019). Self-medication for sleep in college students: Concurrent and prospective associations with sleep and alcohol behavior. *Behavioral Sleep Medicine*, *17*(3), 327-341. https://doi.org/10.1080/15402002.2017.1357119

Goodhines, P. A., Park, A., Gellis, L., Loury, J., & Kim, J. (2016). Substance use for sleep aid in college students: Associations with risky drinking and insomnia severity. *Alcoholism: Clinical and Experimental Research*, *40*(SUPPL. 1), 228A. https://doi.org/10.1111/acer.13084

Gorgich, E. A. C., Moftakhar, L., Barfroshan, S., & Arbabisarjou, A. (2018). Evaluation of internet addiction and mental health among medical sciences students in the southeast of Iran. *Shiraz E Medical Journal*, *19*(1), e55561. https://doi.org/10.5812/semj.55561

Gruezo-Realpe, P., Benavides-Lopez, A., Cedeno-Morejon, R., Noritz-Mero, A., Vinan-Paucar, L., Rosero-Basurto, I., Jaramillo-Castro, M., Chango-Pinargote, A., & Jimenez-Zambrano, J. (2023). Insomnia and associated

factors among medical students recovering from acute COVID-19 infection. *Revista Ecuatoriana de Neurologia*, *32*(2). https://doi.org/10.46997/REVECUATNEUROL32200032

Gruezo-Realpe, P., Chango-Pinargote, A., Jaramillo-Castro, M., Rosero-Basurto, I., Viñan-Paucar, L., Paredes-Gutierrez, E., Jara-Escobar, J., Saona, L. B., & Zambrano, J. J. (2023). Prevalence of Insomnia and Associated Factors Among Medical Students Who Recovered From Acute COVID-19 Infection. *Neurology*, *100*(17), Article 3582. https://doi.org/10.1212/WNL.0000000000203374

Günes, Z., & Arslantas, H. (2017). Insomnia in nursing students and related factors: A cross-sectional study. *International Journal Of Nursing Practice*, *23*(5), Article e12578. https://doi.org/10.1111/ijn.12578

Guo, L., Deng, J. X., He, Y., Deng, X. Q., Huang, J. H., Huang, G. L., Gao, X., & Lu, C. Y. (2014). Prevalence and correlates of sleep disturbance and depressive symptoms among Chinese adolescents: a cross-sectional survey study. *BMJ Open*, *4*(7), Article e005517. https://doi.org/10.1136/bmjopen-2014-005517

Guo, N., Weng, X., Zhao, S. Z., Zhang, J., Wang, M. P., Li, L., & Wang, L. (2023). Adverse childhood experiences on internet gaming disorder mediated through insomnia in Chinese young people. *Frontiers In Public Health*, *11*. https://doi.org/10.3389/fpubh.2023.1283106

Gupta, R., Taneja, N., Anand, T., Gupta, A., Gupta, R., Jha, D., & Singh, S. (2021). Internet Addiction, Sleep Quality and Depressive Symptoms Amongst Medical Students in Delhi, India. *Community Mental Health Journal*, *57*(4), 771-776. https://doi.org/10.1007/s10597-020-00697-2

Gupta, S., Epari, V., & Pradhan, S. (2018). Association of Internet Addiction with Insomnia, Depression, Anxiety and Stress among University Students - A Cross-Sectional Study. *Indian Journal Of Community Health*, *30*(4), 342-347.

Haghighi, M., & Gerber, M. (2019). Does Mental Toughness Buffer the Relationship Between Perceived Stress, Depression, Burnout, Anxiety, and Sleep? *International Journal Of Stress Management*, *26*(3), 297-305. https://doi.org/10.1037/str0000106

Haile, Y. G., Alemu, S. M., & Habtewold, T. D. (2017). Insomnia and Its Temporal Association with Academic Performance among University Students: A Cross-Sectional Study. *BioMed Research International*, *2017*, 1-7. https://doi.org/10.1155/2017/2542367

Hall, C., Poling, K., Athey, A., Alfonso-Miller, P., Gehrels, J., & Grandner, M. A. (2017). Sleep difficulties associated with academic performance in student athletes. *Sleep*, *40*.

Hamilton, J. L., Stange, J. P., Burke, T. A., Franzen, P. L., & Alloy, L. B. (2019). Sleep disturbance and physiological regulation among young adults with prior depression. *Journal of Psychiatric Research*, *115*, 75-81. https://doi.org/10.1016/j.jpsychires.2019.05.016

Hammoudi, S. F., Mreydem, H. W., Ali, B. T. A., Saleh, N. O., Chung, S., Hallit, S., & Salameh, P. (2021). Smartphone screen time among university students in lebanon and its association with insomnia, bedtime procrastination, and body mass index during the covid-19 pandemic: A cross-sectional study. *Psychiatry Investigation*, *18*(9). https://doi.org/10.30773/PI.2021.0120

Hanna, C., & Carter, P. (2013). Sleep and depression in undergraduate college students over time. *Sleep*, *36*(SUPPL. 1).

Hartmann, M. E., & Prichard, J. R. (2018). Calculating the contribution of sleep problems to undergraduates' academic success. *Sleep Health*, *4*(5), 463-471. https://doi.org/10.1016/j.sleh.2018.07.002

Hayer, C. A., & Hicks, R. A. (1993). Type-A-B Scores And Insomnia Among College-Students - A Replication And Extension Of Earlier Studies. *Perceptual and Motor Skills*, *77*(3), 1265-1266. https://doi.org/10.2466/pms.1993.77.3f.1265

Hayley, A. C., Downey, L. A., Stough, C., Sivertsen, B., Knapstad, M., & Overland, S. (2017). Social and emotional loneliness and self-reported difficulty initiating and maintaining sleep (DIMS) in a sample of Norwegian university students. *Scandinavian Journal Of Psychology*, *58*(1), 91-99. https://doi.org/10.1111/sjop.12343

Hayley, A. C., Sivertsen, B., Hysing, M., Vedaa, O., & Overland, S. (2017). Sleep difficulties and academic performance in Norwegian higher education students. *British Journal Of Educational Psychology*, *87*(4), 722-737. https://doi.org/10.1111/bjep.12180

Hendershot, S., Tubbs, A., Fernandez, F. X., Perlis, M., & Grandner, M. (2021). Sleep and non-suicidal self-injury in college students. *Sleep*, *44*(SUPPL 2). https://doi.org/10.1093/sleep/zsab072.774

Hershner, S., Jansen, E., Gavidia, R., Matlen, L., Hoban, M., & Dunietz, G. L. (2021). Associations Between Transgender Identity, Sleep, Mental Health and Suicidality Among a North American Cohort of College Students. *Nature And Science Of Sleep*, *13*. https://doi.org/10.2147/NSS.S286131

Hidalgo, M. P., & Caumo, W. (2002). Sleep disturbances associated with minor psychiatric disorders in medical students. *Neurological Sciences*, *23*(1), 35-39. https://doi.org/10.1007/s100720200021

Hjetland, G. J., Skogen, J. C., Hysing, M., & Sivertsen, B. (2021). The Association Between Self-Reported Screen Time, Social Media Addiction, and Sleep Among Norwegian University Students. *Frontiers in Public Health*, *9*, 794307. https://doi.org/10.3389/fpubh.2021.794307

Hood, H. K., Carney, C. E., & Harris, A. L. (2011). Rethinking safety behaviors in insomnia: Examining the perceived utility of sleep-related safety behaviors. *Behavior Therapy*, *42*(4), 644-654. https://doi.org/10.1016/j.beth.2011.02.004

Hou, G. (2021). Psychological Problems and Intervention Strategies of College Students During COVID-19 Epidemic. *Psychiatria Danubina*, *33*.

Hsu, Y.-W., & Chang, C.-P. (2022). Stress of life events and anxiety as mediators of the association between insomnia and triglycerides in college students. *Journal Of American College Health*, *70*(5), 1396-1402. https://doi.org/10.1080/07448481.2020.1799805

Hussein, O. M., bt Zainal, N. Z., & Abdel-Latif, M. E. (2012). Prevalance of stress among international post-graduate doctors at the University Malaya Medical Centre (UMMC), Kuala Lumpur. *Journal of Clinical and Diagnostic Research*, *6*(5).

Ibrahim, F. M., Salmi, R. N., Saif, M. A., & Mohammed, A. (2024). Sleep Disorders' Prevalence and Impact on Academic Performance among Undergraduate Nursing Students in a Selected University, United Arab Emirates. *Sage Open Nursing*, *10*. https://doi.org/10.1177/23779608241274229

Ionescu, C. G., Chendea, A., & Licu, M. (2023). Is Satisfaction with Online Learning Related to Depression, Anxiety, and Insomnia Symptoms? A Cross-Sectional Study on Medical Undergraduates in Romania.

European Journal Of Investigation In Health Psychology And Education, *13*(3), 580-594. https://doi.org/10.3390/ejihpe13030045

Jain, A., Sharma, R., Gaur, K. L., Yadav, N., Sharma, P., Sharma, N., Khan, N., Kumawat, P., Jain, G., Maanju, M., Sinha, K. M., & Yadav, K. S. (2020). Study of internet addiction and its association with depression and insomnia in university students. *Journal Of Family Medicine And Primary Care*, *9*(3), 1700-1706. https://doi.org/doi:10.4103/jfmpc.jfmpc_1178_19

Jones, R. D., Jackson, W. B., Chang, A. M., Buxton, O. M., & Jackson, C. (2019). Ethnoracial sleep disparities among college students in The United States: A nationally representative study. *Sleep*, *42*(Supplement 1), A65. https://doi.org/10.1093/sleep/zsz067.156

Jose, S., Cyriac, M. C., Sebastian, S., Lidiyamol, P. V., & Dhandapani, M. (2024). Impact of problematic mobile phone use among nursing students in India: Exploring associations with depression, insomnia, self-esteem and satisfaction with life. *International Journal of Nursing Practice*, *30*(5), 1-12. https://doi.org/doi:10.1111/ijn.13247

Joshi, K., Mishra, D., Dubey, H., & Gupta, R. (2015). Sleep pattern and insomnia among medical students: Effect of gender and dysfunctional beliefs and attitudes about sleep. *Somnologie*, *19*(3). https://doi.org/10.1007/s11818-015-0012-x

Kadam, Y. R., Patil, S. R., Waghachavare, V., & Gore, A. D. (2016). Influence of Various Lifestyle and Psychosocial Factors on Sleep Disturbances among the College Students: A Cross-Sectional Study from an Urban Area of India. *Journal Of Krishna Institute Of Medical Sciences University*, *5*(3), 51-60.

Kandeger, A., & Selvi, Y. (2017). Investigation of food addiction and impulsivity relations biological rhythms differences and insomnia in University students. *European Psychiatry*, *41*(Supplement 1). https://doi.org/10.1016/j.eurpsy.2017.02.139

Kang, Y., Liu, S., Yang, L., Xu, B., Lin, L., Xie, L., Zhang, W., Zhang, J., & Zhang, B. (2020). Testing the bidirectional associations of mobile phone addiction behaviors with mental distress, sleep disturbances, and sleep patterns: A one-year prospective study among Chinese college students [Behavior Disorders & Antisocial Behavior 3230]. *Frontiers in Psychiatry*, *11*. https://doi.org/10.3389/fpsyt.2020.00634

Kayaba, M., Matsushita, T., Enomoto, M., Kanai, C., Katayama, N., Inoue, Y., & Sasai-Sakuma, T. (2020). Impact of sleep problems on daytime function in school life: a cross-sectional study involving Japanese university students. *BMC Public Health*, *20*(1), 371-371. https://doi.org/10.1186/s12889-020-08483-1

Khader, W. S., Tubbs, A. S., Haghighi, A., Athey, A. B., Killgore, W. D. S., Hale, L., Perlis, M. L., Gehrels, J.-A., Alfonso-Miller, P., Fernandez, F.-X., & Grandner, M. A. (2020). Onset insomnia and insufficient sleep duration are associated with suicide ideation in university students and athletes. *Journal of Affective Disorders*, *274*, 1161-1164. https://doi.org/10.1016/j.jad.2020.05.102

Khalil, A. H., Saad, A., ElSerafiy, D., Shorub, E., & Hamdy, R. (2023). Stress and Sleep Difficulties in a Sample of Medical Students, Ain-Shams University. *QJM: An International Journal of Medicine*, *116*(Supplement 1). https://doi.org/10.1093/qjmed/hcad069.519

Khan, K., Waqas, M., Sarwar, R., Ahmad, S., & Faizan, M. (2019). Effects of insomnia on daily performance of

medical students: a cross sectional study conducted in university of Lahore, Pakistan. *Rawal Medical Journal*, *44*(3), 622-625.

Khan, W., Badri, H. M., Mohmed, K., Nabag, M. G., Bakri, M., Salih, A., Elamin, M. O., Ekram, R., & Natto, H. A. (2024). A public health view and comparison between online and on-campus learning to evaluate sleep and mental health among undergraduate students. *Journal Of Family Medicine And Primary Care*, *13*(5), 1956-1961. https://doi.org/10.4103/jfmpc.jfmpc_1790_23

Kim, J., Hwang, E. H., Shin, S., & Kim, K. H. (2022). University Students' Sleep and Mental Health Correlates in South Korea. *Healthcare*, *10*(9), Article 1635. https://doi.org/10.3390/healthcare10091635

King, E., Lane, H., & Garland, S. N. (2018). Prevalence and predictive factors of sleeping medication use among students at a Canadian university. *Sleep*, *41*(Supplement 1), A138. https://doi.org/10.1093/sleep/zsy061.360

King, E. R., Willcott Benoit, W., Repa, L. M., & Garland, S. N. (2022). Prevalence and factors associated with non-medical prescription stimulant use to promote wakefulness in young adults [Health Psychology & Medicine 3360]. *Journal of American College Health*, *70*(1), 174-181. https://doi.org/10.1080/07448481.2020.1730851

King, N., Pickett, W., Rivera, D., Byun, J., Li, M., Cunningham, S., & Duffy, A. (2023). The Impact of the COVID-19 Pandemic on the Mental Health of First-Year Undergraduate Students Studying at a Major Canadian University: A Successive Cohort Study. *Canadian Journal of Psychiatry*, *68*(7). https://doi.org/10.1177/07067437221094549

King, N., Rivera, D., Cunningham, S., Pickett, W., Harkness, K., McNevin, S. H., Milanovic, M., Byun, J., Khanna, A., Atkinson, J., Saunders, K. E. A., & Duffy, A. (2023). Mental health and academic outcomes over the first year at university in international compared to domestic Canadian students. *Journal of American College Health*, *71*(9), 2663-2672. https://doi.org/10.1080/07448481.2021.1982950

Kivela, L., Mouthaan, J., van der Does, W., & Antypa, N. (2024). Student mental health during the COVID-19 pandemic: Are international students more affected? *Journal of American College Health*, *72*(2). https://doi.org/10.1080/07448481.2022.2037616

Klainberg, M. B., Roller, M. C., Ambrosio-Mawhirter, D., Johnston, J. P., Hauser, C., Parker, R. D., & Neville, S. M. (2021). Impact of Sleep on Nursing Students in the Era of a Pandemic. *Journal of the New York State Nurses Association*, *48*(1), 57-63.

Kwok, C., Leung, P. Y., Poon, K. Y., & Fung, X. C. C. (2021). The effects of internet gaming and social media use on physical activity, sleep, quality of life, and academic performance among university students in Hong Kong: A preliminary study. *Asian Journal of Social Health and Behavior*, *4*(1), 36-44. https://doi.org/10.4103/shb.shb_81_20

Labiano, L. M., & Fiorentino, M. T. (1996). Psychosomatic disorders in university students. *Revista de Psiquiatria de la Facultad de Medicina de Barcelona*, *23*(5), 152-157.

Lai, A. Y. K., Lee, L., Wang, M. P., Feng, Y., Lai, T. T. K., Ho, L. M., Lam, V. S. F., Ip, M. S. M., & Lam, T. H. (2020). Mental Health Impacts of the COVID-19 Pandemic on International University Students, Related Stressors, and Coping Strategies. *Frontiers in Psychiatry*, *11*, 584240.

https://doi.org/10.3389/fpsyt.2020.584240

Larrabee, K., & Prichard, J. (2016). Prevalence and quality of life correlates of insomnia and other sleep disorders in a national sample of college students. *Sleep*, *39*(SUPPL. 1), A194.

Lawal, A. M., Azikiwe, J. C., & Fadaka, B. F. (2023). School life in COVID-19 post-lockdown: differential effects of socio-demographics and adjustments on mental health indicators among university students. *Psychology, Health & Medicine*, *28*(10). https://doi.org/10.1080/13548506.2023.2192038

Lemma, S., Gelaye, B., Berhane, Y., Worku, A., & Williams, M. A. (2012). Sleep quality and its psychological correlates among university students in Ethiopia: A cross-sectional study [Classroom Dynamics & Student Adjustment & Attitudes 3560]. *BMC Psychiatry*, *12*. https://doi.org/10.1186/1471-244X-12-237

Li, D., & Xiao, R. (2024). Correlation of insomnia with perceived stress in college students. *Journal of Southern Medical University*, *44*(4). https://doi.org/10.12122/j.issn.1673-4254.2024.04.23

Li, F. (2024). The Role of Smartphone Addiction as a Mediator between Psychological Resilience and Insomnia in Medical Students at a University. *Psychiatry and Clinical Psychopharmacology*, *34*(3). https://doi.org/10.5152/pcp.2024.24910

Li, F., Chen, J., Chen, Q., Wang, J., Wang, M., Ma, S., Zhang, B., & Hu, W. (2023). Mental health of junior college students in China during COVID-19 school lockdown: Findings of on-line cross-sectional survey. *Medicine (United States)*, *102*(52), E36808. https://doi.org/10.1097/MD.0000000000036808

Li, H., Zheng, L., Le, H., Zhuo, L., Wu, Q., Ma, G., & Tao, H. (2020). The Mediating Role of Internalized Stigma and Shame on the Relationship between COVID-19 Related Discrimination and Mental Health Outcomes among Back-to-School Students in Wuhan. *International Journal of Environmental Research and Public Health*, *17*(24). https://doi.org/10.3390/ijerph17249237

Li, J. (2024). The relationship between peer support and sleep quality among Chinese college students: the mediating role of physical exercise atmosphere and the moderating effect of eHealth literacy. *Frontiers in Psychology*, *15*, Article 1422026. https://doi.org/10.3389/fpsyg.2024.1422026

Li, J., Luo, C., Liu, L., Huang, A., Ma, Z., Chen, Y., Deng, Y., & Zhao, J. (2024). Depression, anxiety, and insomnia symptoms among Chinese college students: A network analysis across pandemic stages. *Journal of Affective Disorders*, *356*, 54-63. https://doi.org/10.1016/j.jad.2024.04.023

Li, L., Li, X. Y., Li, Y., Liu, X. P., & Huang, L. (2024). Types of short video addiction among college freshmen: Effects on career adaptability, insomnia, and depressive symptoms. *Acta Psychologica*, *248*, Article 104380. https://doi.org/10.1016/j.actpsy.2024.104380

Li, Q., Chen, X., Zhu, Y., & Shi, X. (2024). Developmental pathways from insomnia to suicidality: A resilience perspective. *Journal of Affective Disorders*, *362*, 45-53. https://doi.org/10.1016/j.jad.2024.06.104

Li, S., Fong, D. Y. T., Xu, Y., Wilkinson, K., Shapiro, C., & Wong, J. Y. H. (2021). Measurement properties of the simplified Chinese version of Nonrestorative Sleep Scale in adolescents. *Health & Social Care In The Community*, *29*(6), E299-E307. https://doi.org/10.1111/hsc.13354

Li, T. T., Xie, Y., Tao, S. M., Yang, Y. J., Xu, H. L., Zou, L. W., Tao, F. B., & Wu, X. Y. (2020). Chronotype, Sleep, and Depressive Symptoms Among Chinese College Students: A Cross-Sectional Study. *Frontiers in*

Neurology, *11*, Article 592825. https://doi.org/10.3389/fneur.2020.592825

Li, Y., Bai, W., Zhu, B., Duan, R., Yu, X., Xu, W., Wang, M., Hua, W., Yu, W., Li, W., & Kou, C. (2020). Prevalence and correlates of poor sleep quality among college students: a cross-sectional survey. *Health & Quality of Life Outcomes*, *18*(1), 1-11. https://doi.org/10.1186/s12955-020-01465-2

Li, Y., Qin, Q., & Zheng, D. (2022). Short sleep duration is associated with suicidal ideation during the COVID-19 outbreak in medical students: a longitudinal cohort study. *Sleep Medicine*, *100*(Supplement 1), S89. https://doi.org/10.1016/j.sleep.2022.05.249

Liang, S.-w., Liu, L.-l., Peng, X.-d., Chen, J.-b., Huang, A.-d., Wang, X.-y., Zhao, J.-b., Fan, F., & Liu, X.-c. (2022). Prevalence and associated factors of suicidal ideation among college students during the COVID-19 pandemic in China: A 3-wave repeated survey. *BMC Psychiatry*, *22*. https://doi.org/10.1186/s12888-022-03968-2

Liang, X., Zhang, H., Wang, X., Li, D., Liu, Y., & Qiu, S. (2025). Longitudinal neurofunctional alterations following nonpharmacological treatments and the mediating role of regional homogeneity in subclinical depression comorbid with sleep disorders among college students. *Journal of Psychiatric Research*, *181*. https://doi.org/10.1016/j.jpsychires.2024.12.038

Liang, Y., Si, G. W., Hu, H. J., Zhang, Z. W., Song, C. P., Dou, Q. F., & Wen, J. G. (2022). Prevalence, Risk Factors, and Psychological Effects of Overactive Bladder in Chinese University Students. *International Neurourology Journal*, *26*(4). https://doi.org/10.5213/INJ.2244188.094

Liao, W., Luo, X., Kong, F., Sun, Y., & Ye, Z. (2024). Association between non-restorative sleep and psychotic-like experiences among Chinese college students: A latent profile and moderated mediation analysis. *Schizophrenia Research*, *270*, 295-303. https://doi.org/10.1016/j.schres.2024.06.038

Lindsay, J. A. B., McGowan, N. M., King, N., Rivera, D., Li, M., Byun, J., Cunningham, S., Saunders, K. E. A., & Duffy, A. (2022). Psychological predictors of insomnia, anxiety and depression in university students: potential prevention targets. *BJPsych Open*, *8*(3), e86. https://doi.org/10.1192/bjo.2022.48

Liu, S., Wing, Y. K., Hao, Y., Li, W., Zhang, J., & Zhang, B. (2019). The associations of long-time mobile phone use with sleep disturbances and mental distress in technical college students: A prospective cohort study. *Sleep: Journal of Sleep and Sleep Disorders Research*, *42*(2), 1-10. https://doi.org/10.1093/sleep/zsy213

Liu, X., Peng, X., Guo, C., Wu, H., Liu, D., & Li, C. (1995). Insomnia in young students and associated factors. *Chinese Journal of Clinical Psychology*, *3*(4), 230-232.

Liu, X. C., Uchiyama, M., Okawa, M., & Kurita, H. (2000). Prevalence and correlates of self-reported sleep problems among Chinese adolescents. *Sleep*, *23*(1), 27-34.

Liu, Y. L., Chen, J. J., Chen, K., Liu, J., & Wang, W. (2023). The associations between academic stress and depression among college students: A moderated chain mediation model of negative affect, sleep quality, and social support. *Acta Psychologica*, *239*, Article 104014. https://doi.org/10.1016/j.actpsy.2023.104014

Liu, Z., Liu, R. X., Zhang, Y., Zhang, R., Liang, L. J., Wang, Y., Wei, Y. G., Zhu, R. X., & Wang, F. (2021). Association between perceived stress and depression among medical students during the outbreak of COVID-19:

The mediating role of insomnia. *Journal of Affective Disorders*, *292*, 89-94. https://doi.org/10.1016/j.jad.2021.05.028

Loayza H, M. P., Ponte, T. S., Carvalho, C. G., Pedrotti, M. R., Nunes, P. V., Souza, C. M., Zanette, C. B., Voltolini, S., & Chaves, M. L. (2001). Association between mental health screening by self-report questionnaire and insomnia in medical students. *Arquivos De Neuro-Psiquiatria*, *59*(2-A), 180-185. https://doi.org/10.1590/s0004-282x2001000200005

Lohsoonthorn, V., Khidir, H., Casillas, G., Lertmaharit, S., Tadesse, M. G., Pensuksan, W. C., Rattananupong, T., Gelaye, B., & Williams, M. A. (2013). Sleep quality and sleep patterns in relation to consumption of energy drinks, caffeinated beverages, and other stimulants among Thai college students. *Sleep & breathing = Schlaf & Atmung*, *17*(3), 1017-1028. https://doi.org/10.1007/s11325-012-0792-1

Loke, Y. M., Lim, S. J. Y., Rukmini, A. V., Sumarta, T. T., Chen, P., Wang, C. K. J., & Gooley, J. J. (2022). Sleep Health And Wellness Questionnaire (SHAWQ) scores associate with sleep problems, depression symptoms, and academic performance in adolescents and university students. *Journal of Sleep Research*, *31*(Supplement 1). https://doi.org/10.1111/jsr.13740

Lombardo, C. (2012). The role of emotion regulation in the relationship between insomnia and eating disorders. *Journal of Sleep Research*, *21*(SUPPL. 1), 107. https://doi.org/10.1111/j.1365-2869.2012.01044.x

Lombardo, C., Battagliese, G., Baglioni, C., David, M., Violani, C., & Riemann, D. (2014). Severity of insomnia, disordered eating symptoms, and depression in female university students. *Clinical Psychologist*, *18*(3), 108-115. https://doi.org/10.1111/cp.12023

Low, B. S., Koshy, S., Thein, K. M. M., Tayeba, S., & Saha, S. (2024). Factors Associated with Sleep Disorders Among International University Students in Malaysia. *Sleep and Vigilance*, *8*(1). https://doi.org/10.1007/s41782-023-00259-y

Lukowski, A. F., Kamliot, D. Z., & Schlaupitz, C. A. (2024). Insomnia and behaviorally induced sleep syndrome in undergraduates tested during the COVID-19 pandemic: associations with health, stress, and GPA. *Journal of Clinical Sleep Medicine*, *20*(2). https://doi.org/10.5664/jcsm.10844

Lukowski, A. F., & Tsukerman, D. (2021). Temperament, sleep quality, and insomnia severity in university students: Examining the mediating and moderating role of sleep hygiene. *PLoS One*, *16*(7), e0251557. https://doi.org/10.1371/journal.pone.0251557

Lund, H. G., Reider, B. D., Whiting, A. B., & Prichard, J. R. (2010). Sleep patterns and predictors of disturbed sleep in a large population of college students. *Journal of Adolescent Health*, *46*(2), 124-132. https://doi.org/10.1016/j.jadohealth.2009.06.016

Ma, S., Yang, J., Xu, J., Zhang, N., Kang, L., Wang, P., Wang, W., Yang, B., Li, R., Xiang, D., Bai, H., & Liu, Z. (2022). Using network analysis to identify central symptoms of college students' mental health. *Journal of Affective Disorders*, *311*, 47-54. https://doi.org/10.1016/j.jad.2022.05.065

Ma, Z., Wang, D., Zhao, J., Zhu, Y., Zhang, Y., Chen, Z., Jiang, J., Pan, Y., Yang, Z., Zhu, Z., Liu, X., & Fan, F. (2022). Longitudinal associations between multiple mental health problems and suicidal ideation among university students during the COVID-19 pandemic [Psychological & Physical Disorders 3200]. *Journal*

of Affective Disorders, *311*, 425-431. https://doi.org/10.1016/j.jad.2022.05.093

Madsen, J. W., Hernandez, L., Sedov, I., & Tomfohr-Madsen, L. M. (2023). Romantic relationship satisfaction is associated with sleep in undergraduate students [Group & Interpersonal Processes 3020]. *Couple and Family Psychology: Research and Practice*, *12*(1), 39-54. https://doi.org/10.1037/cfp0000163

Mahfouz, M. S., Ali, S. A., Bahari, A. Y., Ajeebi, R. E., Sabei, H. J., Somaily, S. Y., Madkhali, Y. A., Hrooby, R. H., & Shook, R. N. (2020). Association Between Sleep Quality and Physical Activity in Saudi Arabian University Students. *Nature And Science Of Sleep*, *12*, 775-782. https://doi.org/10.2147/NSS.S267996

Makhal, M., Ray, P. K., Ray, S., Ghosh, S., Majumder, U., De, S., Bandyopadhyay, G. K., & Bera, N. K. (2015). Prevalence of psychiatric morbidity among undergraduate students of a dental college in west bengal. *Journal of Clinical and Diagnostic Research*, *9*(7). https://doi.org/10.7860/JCDR/2015/13642.6230

Mansour, T. M. A., & Yousef, M. (2016). Nightmares among young medical students. *Biomedical Research (India)*, *27*(2).

Manzar, D. (2020). Insomnia and Related Complaints: Association with chronic disease, mild-severe levels of anxiety and poor sleep hygiene in Ethiopian collegiate young adults. *Pakistan Journal Of Medical & Health Sciences*, *14*(4), 1769-1774.

Manzar, M. D., Noohu, M. M., Salahuddin, M., Nureye, D., Albougami, A., Spence, D. W., Pandi-Perumal, S. R., & BaHammam, A. S. (2020). Insomnia Symptoms and Their Association with Anxiety and Poor Sleep Hygiene Practices Among Ethiopian University Students. *Nature And Science Of Sleep*, *12*, 575-582. https://doi.org/10.2147/NSS.S246994

Manzar, M. D., Salahuddin, M., Alamri, M., Albougami, A., Khan, M. Y. A., Nureye, D., Spence, D. W., & Pandi-Perumal, S. R. (2019). Psychometric properties of the Epworth sleepiness scale in Ethiopian university students. *Health and Quality of Life Outcomes*, *17*(1), 30. https://doi.org/10.1186/s12955-019-1098-9

Manzar, M. D., Salahuddin, M., Pandi-Perumal, S. R., & Bahammam, A. S. (2021). Insomnia May Mediate the Relationship Between Stress and Anxiety: A Cross-Sectional Study in University Students. *Nature And Science Of Sleep*, *13*, 31-38. https://doi.org/10.2147/NSS.S278988

Marta, O. F. D., Kuo, S.-Y., Bloomfield, J., Lee, H.-C., Ruhyanudin, F., Poynor, M. Y., Brahmadhi, A., Pratiwi, I. D., Aini, N., Mashfufa, E. W., Hasan, F., & Chiu, H.-Y. (2020). Gender differences in the relationships between sleep disturbances and academic performance among nursing students: A cross-sectional study. *Nurse Education Today*, *85*. https://doi.org/10.1016/j.nedt.2019.104270

Matsuda, E., & Kikutani, M. (2022). The Interactive Influence of Life Stressor and Sleep Disturbance on Depression: A Cross-Sectional Examination on Chinese and Japanese University Students. *Sage Open*, *12*(1). https://doi.org/10.1177/21582440221086610

Mbous, Y. P. V., Nili, M., Mohamed, R., & Dwibedi, N. (2022). Psychosocial Correlates of Insomnia Among College Students. *Preventing Chronic Disease*, *19*, 1-10. https://doi.org/10.5888/pcd19.220060

Meaklim, H., Le, F., Drummond, S. P. A., Bains, S. K., Varma, P., Junge, M. F., & Jackson, M. L. (2024). Insomnia is more likely to persist than remit after a time of stress and uncertainty: a longitudinal cohort study examining trajectories and predictors of insomnia symptoms. *Sleep*, *47*(4). https://doi.org/10.1093/sleep/zsae028

Meaklim, H., Meltzer, L. J., Rehm, I. C., Junge, M. F., Monfries, M., Kennedy, G. A., Bucks, R. S., Graco, M., & Jackson, M. L. (2023). Disseminating sleep education to graduate psychology programs online: A knowledge translation study to improve the management of insomnia [Professional Education & Training 3410]. *Sleep: Journal of Sleep and Sleep Disorders Research*, *46*(10), 1-17. https://doi.org/10.1093/sleep/zsad169

Meaklim, H., Rehm, I. C., Monfries, M., Junge, M., Meltzer, L. J., & Jackson, M. L. (2021). Wake up psychology! Postgraduate psychology students need more sleep and insomnia education [Professional Education & Training 3410]. *Australian Psychologist*, *56*(6), 485-498. https://doi.org/10.1080/00050067.2021.1955614

Medina-Ortiz, O., Cardenas, C., Arenas-Villamizar, V., Riano-Garzon, M., & Bermudez-Pirela, V. (2024). Insomnia and stress during the COVID-19 pandemic in psychology students from Cucuta, Colombia. *Sleep Medicine*, *115*(Supplement 1). https://doi.org/10.1016/j.sleep.2023.11.507

Mendoza, L., Marco, G., Gonzalo, C. V., Ali, A. K. C., Rosales, K. A. A., Chavez, R., Bruno, S., & Alvarado, G. F. (2021). Use of energy drinks and insomnia symptoms in medical students from a Peruvian university. *Revista Chilena de Neuro-Psiquiatria*, *59*(4), 289-301. https://doi.org/10.4067/S0717-92272021000400289

Meng, J., Wang, F., Chen, R. T., Hua, H., Yang, Q., Yang, D., Wang, N., Li, X., Ma, F. F., Huang, L. T., Zou, Z. Z., Li, M. L., Wang, T. T., Luo, Y. N., Li, Y. D., & Liu, Y. (2021). Association between the pattern of mobile phone use and sleep quality in Northeast China college students. *Sleep and Breathing*, *25*(4), 2259-2267. https://doi.org/10.1007/s11325-021-02295-2

Merchan Tamayo, J. P., Rocchi, M. A., St-Denis, B., Bonneville, L., & Beaudry, S. G. (2024). A motivational approach to understanding problematic smartphone use and negative outcomes in university students. *Addictive Behaviors*, *148*, 107842. https://doi.org/10.1016/j.addbeh.2023.107842

Meyer-Szary, J., Jakitowicz, M., Sieczkowski, M., Kasperczyk, J., & Jakitowicz, J. (2008). The quality of sleep in college students Tricity. *Sen*, *8*(1), 15-21.

Miadich, S., & Tagler, M. (2011). Sleep problems in a college population: What role circadian rhythms may play and relations to depression and anxiety. *Sleep*, *34*(SUPPL. 1), A166.

Michaeli, D., Keough, G., Strotzer, Q., & Michaeli, T. (2022). Digital medical education and students' mental health: Effects of the COVID-19 pandemic in Germany [Professional Education & Training 3410

Health & Mental Health Services 3370]. *The Journal of Mental Health Training, Education and Practice*, *17*(4), 305-322. https://doi.org/10.1108/JMHTEP-03-2021-0035

Milicev, J., McCann, M., Simpson, S. A., Biello, S. M., & Gardani, M. (2023). Evaluating Mental Health and Wellbeing of Postgraduate Researchers: Prevalence and Contributing Factors. *Current Psychology*, *42*(14), 12267-12280. https://doi.org/10.1007/s12144-021-02309-y

Miller, M. B., Yurasek, A. M., Pritshmann, R., Curtis, A. F., & Mc Crae, C. S. (2019). Negative mood as a mediator of the association between insomnia symptoms and marijuana problems in young adults. *Sleep*, *42*(Supplement 1), A136. https://doi.org/10.1093/sleep/zsz067.331

Milojevich, H. M., & Lukowski, A. F. (2016). Sleep and Mental Health in Undergraduate Students with Generally

Healthy Sleep Habits. *PLoS One*, *11*(6), Article e0156372. https://doi.org/10.1371/journal.pone.0156372

Moayedi, F., Shahabjahanlu, A., Rasa, F., & Sadeghi, P. (2015). Prevalence of Sleep Disorders among Medical Students. *Research Journal Of Pharmaceutical Biological And Chemical Sciences*, *6*(2), 894-898.

Moghaddam, J. F., Nakhaee, N., Sheibani, V., Garrusi, B., & Amirkafi, A. (2012). Reliability and validity of the Persian version of the Pittsburgh Sleep Quality Index (PSQI-P). *Sleep and Breathing*, *16*(1), 79-82. https://doi.org/10.1007/s11325-010-0478-5

Mohamed, E. Y., Abdulrahim, S. A., Sami, W., Althaqib, A. N., Alzuwayyid, A. A., Almutiri, K. A., AlAbdulmunim, A. M., & Alhokel, K. H. (2020). Insomnia and Related Anxiety Among Medical Students. *Journal Of Research In Medical And Dental Science*, *8*(3), 198-202.

Mohammadbeigi, A., Absari, R., Valizadeh, F., Saadati, M., Sharifimoghadam, S., Ahmadi, A., Mokhtari, M., & Ansari, H. (2016). Sleep Quality in Medical Students; the Impact of Over-Use of Mobile Cell-Phone and Social Networks. *Journal Of Research In Health Sciences*, *16*(1), 46-50.

Moked, Z., Tzischinsky, O., & Shochat, T. (2016). Predisposing factors associated with insomnia in nursing and social sciences college students. *Journal of Sleep Research*, *25*(Supplement 1). https://doi.org/10.1111/jsr.12446

Moo Estrella, J., Rosado Narvaez, C., Yanez oria, A., & Valencia Flores, M. (2013). Types of insomnia and physical activity in college students. *Sleep Medicine*, *14*(SUPPL. 1), e117. https://doi.org/10.1016/j.sleep.2013.11.260

Moo-Estrella, J., Pérez-Benítez, H., Solís-Rodríguez, F., & Arankowsky-Sandoval, G. (2005). Evaluation of depressive symptoms and sleep alterations in college students. *Archives Of Medical Research*, *36*(4), 393-398. https://doi.org/10.1016/j.arcmed.2005.03.018

Mousavi, Z. A., Lai, J., Simon, K., Rivera, A. P., Yunusova, A., Hu, S. R., Labbaf, S., Jafarlou, S., Dutt, N. D., Jain, R. C., Rahmani, A. M., & Borelli, J. L. (2022). Sleep Patterns and Affect Dynamics Among College Students During the COVID-19 Pandemic: Intensive Longitudinal Study. *JMIR Formative Research*, *6*(8), Article e33964. https://doi.org/10.2196/33964

Moussa-Chamari, I., Farooq, A., Romdhani, M., Washif, J. A., Bakare, U., Helmy, M., Al-Horani, R. A., Salamh, P., Robin, N., & Hue, O. (2024). The relationship between quality of life, sleep quality, mental health, and physical activity in an international sample of college students: a structural equation modeling approach. *Frontiers In Public Health*, *12*, 1397924. https://doi.org/10.3389/fpubh.2024.1397924

Moussa-Chamari, I., Romdhani, M., Farooq, A., Trabelsi, K., Yousfi, N., Kamoun, K., Ahmadian, A., Almarri, S. S., Chamari, K., & Hue, O. (2024). Exploring Sleep Patterns in 3,475 College Students: A Comparative Study of Geographical Location, Gender, and Age. *Sleep Science*. https://doi.org/10.1055/s-0044-1788288

Mozaffari, A. (2016). Insomnia and sleep disturbance independent risk factors and its chronotype correlates. *Sleep*, *39*(SUPPL. 1), A179.

Muanprasong, S., & Taneepanichskul, N. (2017). Sleep Quality And Migraine Status Among Undergraduate Students In A Large Urban University Thailand. *Journal Of Health Research*, *31*, S25-S31.

https://doi.org/10.14456/jhr.2017.64

Munezawa, T., Kaneita, Y., Yokoyama, E., Suzuki, H., & Ohida, T. (2009). Epidemiological study of nightmare and sleep paralysis among Japanese adolescents. *Sleep and Biological Rhythms*, *7*(3), 201-210. https://doi.org/10.1111/j.1479-8425.2009.00404.x

Nadeem, A., Cheema, M. K., Naseer, M., & Javed, H. (2018). Comparison of quality of sleep between medical and non-medical undergraduate Pakistani students. *JPMA. The Journal of the Pakistan Medical Association*, *68*(10), 1465-1470.

Nadorff, M. R., Nazem, S., & Fiske, A. (2013). Insomnia symptoms, nightmares, and suicide risk: Duration of sleep disturbance matters [Physical & Somatic Disorders 3290]. *Suicide And Life-Threatening Behavior*, *43*(2), 139-149. https://doi.org/10.1111/sltb.12003

Nadorff, M. R., Salem, T., Winer, E. S., Lamis, D. A., Nazem, S., & Berman, M. E. (2014). Explaining alcohol use and suicide risk: a moderated mediation model involving insomnia symptoms and gender. *Journal Of Clinical Sleep Medicine*, *10*(12), 1317-1323. https://doi.org/10.5664/jcsm.4288

Nadorff, M. R., Winer, E., Chaney, L. D., Salem, T., & Harris, H. (2013). Examining the association of insomnia symptoms, nightmares, and suicidal ideation in relation to the interpersonal-psychological theory of suicide. *Sleep*, *36*(SUPPL. 1), A314.

Nagasaki, K., Kobayashi, H., Nishizaki, Y., Kurihara, M., Watari, T., Shimizu, T., Yamamoto, Y., Shikino, K., Fukui, S., Nishiguchi, S., Katayama, K., & Tokuda, Y. (2024). Association of sleep quality with duty hours, mental health, and medical errors among Japanese postgraduate residents: a cross-sectional study. *Scientific Reports*, *14*(1), 1481. https://doi.org/10.1038/s41598-024-51353-8

Nagose, V., Soni, D., Rathod, S., & Yelne, Y. (2021). COVID-19 impact on mental health, sleep quality and various aspects of life of medical students and interns, and nonmedical students: A comparative study in Indian scenario. *Annals Of Indian Psychiatry*, *5*(2), 158-163. https://doi.org/10.4103/aip.aip_79_21

Nam, H., Chang, J., Manber, R., Trockel, M., Okajima, I., Yang, C. M., Li, S., & Suh, S. (2021). Predictors of dropout in university students participating in an 8-week e-mail based cognitive behavioral therapy for insomnia. *Sleep*, *44*(SUPPL 2). https://doi.org/10.1093/sleep/zsab072.365

Navarro-Martinez, R., Chover-Sierra, E., Colomer-Perez, N., Vlachou, E., Andriuseviciene, V., & Cauli, O. (2020). Sleep quality and its association with substance abuse among university students. *Clinical Neurology and Neurosurgery*, *188*, 105591. https://doi.org/10.1016/j.clineuro.2019.105591

Nikolaev, E. L., Nikolaeva, T., & Alhasan, M. (2024). Sleep disorders among university students as underestimated mental health problem. *European Psychiatry*, *67*(Supplement 1). https://doi.org/10.1192/j.eurpsy.2024.1609

Nisarga, V., Anupama, M., & Kulkarni, H. (2018). Stress and insomnia among first year post graduate residents. *Indian Journal of Psychiatry*, *60*(5 Supplement 1), S94. (70th Annual National Conference of Indian Psychiatric Society, ANCIPS 2018. Ranchi India.)

Nojomi, M., Bandi, M. F. G., & Kaffashi, S. (2009). Sleep Pattern in Medical Students and Residents. *Archives Of Iranian Medicine*, *12*(6), 542-549.

Nsengimana, A., Mugabo, E., Niyonsenga, J., Hategekimana, J. C., Biracyaza, E., Mutarambirwa, R., Ngabo, E., &

Nduwayezu, R. (2023). Sleep quality among undergraduate medical students in Rwanda: a comparative study. *Scientific Reports*, *13*(1), 265. https://doi.org/10.1038/s41598-023-27573-9

Nugent, K., Raj, R., & Nugent, R. (2020). Sleep Patterns and Health Behaviors in Healthcare Students. *Southern Medical Journal*, *113*(3), 104-110. https://doi.org/10.14423/SMJ.0000000000001077

Oliveira, J. C., Neves, V. R., Cespedes, J. G., D'Almeida, V., Okuno, M. F. P., Figueiredo, L. L., Cerqueira, A. R. B., & Rosa, A. D. S. (2024). Predisposing factors for symptoms of anxiety, depression, and insomnia in university students. *Revista Brasileira De Enfermagem*, *77*(6), e20230387. https://doi.org/10.1590/0034-7167-2023-0387

Pacella, K. A. C., Richson, B. N., Short, N. A., Bottera, A. R., Irish, L. A., Perko, V. L., & Forbush, K. T. (2024). Using item response theory to identify key symptoms of insomnia in a sample of university students with probable eating disorders. *Eating And Weight Disorders : EWD*, *29*(1), 49. https://doi.org/10.1007/s40519-024-01679-z

Pallesen, S., Bjorvatn, B., Nordhus, I. H., Sivertsen, B., Hjornevik, M., & Morin, C. M. (2008). A new scale for measuring insomnia: the Bergen insomnia scale. *Perceptual and Motor Skills*, *107*(3), 691-706. https://doi.org/10.2466/PMS.107.3.691-706

Paudel, K., Shah, S., Khanal, P., Bhattarai, M., Gautam, K., Bhusal, S., Bhandari, P., Pandey, A., Niroula, S., Adhikari, T. B., & Shrestha, R. (2024). Association of sleep quality, smartphone addiction, and depressive symptoms among undergraduate medical students in Nepal. *Discover Psychology*, *4*(1). https://doi.org/10.1007/s44202-024-00246-y

Pavlova, I., & Rogowska, A. M. (2023). Exposure to war, war nightmares, insomnia, and war-related posttraumatic stress disorder: A network analysis among university students during the war in Ukraine [Psychological & Physical Disorders 3200]. *Journal of Affective Disorders*, *342*, 148-156. https://doi.org/10.1016/j.jad.2023.09.003

Pervez, S. A., Kumar, H., Bai, S., Kumar, R., Parkash, O., & Avinash. (2021). Prevalence of Insomnia Among Medical Students. *Pakistan Journal Of Medical & Health Sciences*, *15*(4), 1228-1230.

Petrie, K. A., Messman, B. A., Slavish, D. C., Moore, E. W. G., & Petrie, T. A. (2023). Sleep disturbances and depression are bidirectionally associated among college student athletes across COVID-19 pandemic exposure classes. *Psychology Of Sport And Exercise*, *66*, Article 102393. https://doi.org/10.1016/j.psychsport.2023.102393

Petrov, M. E., Lichstein, K. L., & Baldwin, C. M. (2014). Prevalence of sleep disorders by sex and ethnicity among older adolescents and emerging adults: Relations to daytime functioning, working memory and mental health [Physical & Somatic Disorders 3290]. *Journal of Adolescence*, *37*(5), 587-597. https://doi.org/10.1016/j.adolescence.2014.04.007

Piotrowski, P., Babicki, M., Maslanka, P., Kotowicz, K., Marciniak, D., & Rymaszewska, J. (2021). Assessment of sleep disorders and use of psychoactive drugs among Polish students. *Psychiatria Polska*, *54*(5). https://doi.org/10.12740/PP/111751

Poorolajal, J., Ghaleiha, A., Darvishi, N., Daryaei, S., & Panahi, S. (2017). The Prevalence of Psychiatric Distress and Associated Risk Factors among College Students Using GHQ-28 Questionnaire. *Iranian Journal Of*

Public Health, *46*(7), 957-963.

Powell, R., & Nielsen, T. (2019). The relationship of nightmares, insomnia, cataplexy, mirror behaviors, and psychological distress to suicidal ideation in undergraduate students. *Sleep Medicine*, *64*(Supplement 1), S307. https://doi.org/10.1016/j.sleep.2019.11.861

Qeadan, F., Beaudin, S., Reutrakul, S., & English, K. (2024). Single use of psychoactive substances and its association with sleep disorders and sleep health in a large US college sample. *Journal of American College Health*. https://doi.org/10.1080/07448481.2024.2317171

Qiao, X., Shi, X., Chen, X., & Zhu, Y. (2023). Associations between insomnia symptom trajectories with depression and self-harm behaviors in Chinese college students before and during the COVID-19 pandemic: A five-wave longitudinal investigation [Psychological & Physical Disorders 3200]. *Journal of Affective Disorders*, *339*, 877-886. https://doi.org/10.1016/j.jad.2023.07.108

Ramirez, S., Valdes, J., Diaz, F., Solorza, F., Christiansen, P., Lorca, G., & Gaete, J. (2022). Mental health and associated factors among undergraduate students during Covid-19 pandemic in Chile. *European Psychiatry*, *65*(Supplement 1). https://doi.org/10.1192/j.eurpsy.2022.861

Ramon-Arbues, E., Gea-Caballero, V., Granada-Lopez, J. M., Juarez-Vela, R., Pellicer-Garcia, B., & Anton-Solanas, I. (2020). The prevalence of depression, anxiety and stress and their associated factors in college students. *International Journal of Environmental Research and Public Health*, *17*(19). https://doi.org/10.3390/ijerph17197001

Ramón-Arbués, E., Granada-López, J. M., Martínez-Abadía, B., Echaniz-Serrano, E., Antón-Solanas, I., & Jerue, B. A. (2022). The Association between Diet and Sleep Quality among Spanish University Students. *Nutrients*, *14*(16), Article 3291. https://doi.org/10.3390/nu14163291

Ramos, J. N., Muraro, A. P., Nogueira, P. S., Ferreira, M. G., & Rodrigues, P. R. M. (2021). Poor sleep quality, excessive daytime sleepiness and association with mental health in college students. *Annals Of Human Biology*, *48*(5), 382-388. https://doi.org/10.1080/03014460.2021.1983019

Rani, S., Sinha, N., & Kumar, R. (2024). Prevalence of internet addiction and its relationship with insomnia, depression, anxiety, and stress among medical students of a tertiary care medical institute of Eastern India. *Industrial Psychiatry Journal*, *33*(1), 94-100. https://doi.org/10.4103/ipj.ipj_134_23

Rayzah, M., Sami, W., Almutairi, G. A., Almulhim, Y. A., Almuqren, N. M., Aldafas, H. A., Aljameeli, A. G., & Albaqami, F. M. (2021). The Effect of Smartphone Usage at Bedtime and its Relationship with Insomnia and Academic Performance among Majmaah University Students. *Journal Of Research In Medical And Dental Science*, *9*(1), 37-42.

Regestein, Q., Natarajan, V., Pavlova, M., Kawasaki, S., Gleason, R., & Koff, E. (2010). Sleep debt and depression in female college students. *Psychiatry Research*, *176*(1), 34-39. https://doi.org/10.1016/j.psychres.2008.11.006

Repa, L. M., Rodriguez, N., & Garland, S. N. (2018). Power off is better off: The impact of technology use on sleep among university students. *Sleep*, *41*(Supplement 1), A140.

Rezaei, M., Khormali, M., Akbarpour, S., Sadeghniiat-Hagighi, K., & Shamsipour, M. (2018). Sleep quality and its association with psychological distress and sleep hygiene: a cross-sectional study among pre-clinical

medical students. *Sleep Science*, *11*(4), 274-280. https://doi.org/10.5935/1984-0063.20180043

Riaz, M., Rabbani, M. H., Sabir, M. J., Khan, M. A., Manzoor, N., & Chaudhry, M. N. (2022). Predictors of sleep paralysis and relationship of sleep paralysis with sleep quality in university students of Islamabad. *Journal of the Pakistan Medical Association*, *72*(3). https://doi.org/10.47391/JPMA.1313

Ristovska, G., Manusheva, N., Aleksovski, B., Bukovetz, J., Stojanovska, V., & Babinkostova, Z. (2023). Depression and insomnia among students during the COVID-19 pandemic-a cross sectional study. *Srpski Arhiv Za Celokupno Lekarstvo*, *151*(5-6), 278-284. https://doi.org/10.2298/SARH230324052R

Rosenberg, L., Rigney, G., Jemcov, A., van Voorst, D., & Corkum, P. (2024). Usability of an eHealth sleep education intervention for university students. *Digital Health*, *10*, Article 20552076241260480. https://doi.org/10.1177/20552076241260480

Ruiter, M. E., & Lichstein, K. L. (2012). Ethnic disparities in sleep disorders and daytime functioning amongst college students. *Sleep*, *35*(SUPPL. 1), A237. (26th Annual Meeting of the Associated Professional Sleep Societies, LLC, SLEEP 2012. Boston, MA United States.)

Ruivo Marques, D., Gomes, A. A., & Gellis, L. A. (2019). Insomnia in College Students: Specificities Regarding Psychological Treatment. *Sleep & Hypnosis*, *21*(3), 175-187. https://doi.org/10.5350/Sleep.Hypn.2019.21.0186

Rutigliano, J. V., Mindell, J. A., & DuMond, C. (2011). Sleep habits and insomnia symptoms in college students. *Sleep*, *34*(SUPPL. 1), A70. (25th Anniversary Meeting of the Associated Professional Sleep Societies, LLC, SLEEP 2011. Minneapolis, MN United States.)

Sadigh, M. R., Himmanen, S. A., & Scepansky, J. A. (2014). An investigation of the prevalence of insomnia in college students and its relationship to trait anxiety [Physical & Somatic Disorders 3290]. *College Student Journal*, *48*(3), 397-406.

Samaranayake, C., Arroll, B., & Fernando, A. (2014). Sleep disorders among tertiary students: A cross sectional study. *Sleep and Biological Rhythms*, *12*(SUPPL. 1), 39. https://doi.org/10.1111/sbr.12082

Samaranayake, C. B., Arroll, B., & Fernando 3rd, A. T. (2014). Sleep disorders, depression, anxiety and satisfaction with life among young adults: a survey of university students in Auckland, New Zealand. *New Zealand Medical Journal*, *127*(1399), 13-22.

Sánchez-Oviedo, S., Solarte-Rodríguez, W. E., Correa-Carlosama, S. T., Imbachí-Quinayás, D. Y., Anaya-Ramírez, M. L., Mera-Mamián, A. Y., Paz-Peña, C. I., & Zúñiga-Pino, A. (2021). Related factors with sleep quality and daytime sleepiness in university students from the south-west of Colombia. *Duazary*, *18*(3), 232-246. https://doi.org/10.21676/2389783X.4230

Sasaki, M. (2007). Barriers to use of mental health services by Japanese university students. *Psychological Reports*, *100*(2). https://doi.org/10.2466/PR0.100.2.400-406

Schepis, T. S., De Nadai, A. S., Bravo, A. J., Looby, A., Villarosa-Hurlocker, M. C., & Earleywine, M. (2021). Alcohol use, cannabis use, and psychopathology symptoms among college students before and after COVID-19 [Psychological Disorders 3210]. *Journal of Psychiatric Research*, *142*, 73-79. https://doi.org/10.1016/j.jpsychires.2021.07.040

Schlarb, A., & Grunwald, J. (2018). Insomnia, nightmares and daytime sleepiness in university students with

ADHD. *Journal of Sleep Research*, *27*(Supplement 1). https://doi.org/10.1111/jsr.12751

Schlarb, A. A., Classen, M., Grünwald, J., & Vögele, C. (2017). Sleep disturbances and mental strain in university students: results from an online survey in Luxembourg and Germany. *International Journal of Mental Health Systems*, *11*, Article 24. https://doi.org/10.1186/s13033-017-0131-9

Schmidt, R. E., Gay, P., Ghisletta, P., & Van Der Linden, M. (2010). Linking impulsivity to dysfunctional thought control and insomnia: a structural equation model. *Journal of Sleep Research*, *19*(1 Pt 1), 3-11. https://doi.org/10.1111/j.1365-2869.2009.00741.x

Schmidt, R. E., Gay, P., & Van der Linden, M. (2008). Facets of impulsivity are differentially linked to insomnia: Evidence from an exploratory study [Physical & Somatic Disorders 3290]. *Behavioral Sleep Medicine*, *6*(3), 178-192. https://doi.org/10.1080/15402000802162570

Scotta, A. V., Cortez, M. V., & Miranda, A. R. (2022). Insomnia is associated with worry, cognitive avoidance and low academic engagement in Argentinian university students during the COVID-19 social isolation [Health Psychology & Medicine 3360]. *Psychology, Health & Medicine*, *27*(1), 199-214. https://doi.org/10.1080/13548506.2020.1869796

Seehuus, M., Carr, M., Rodriguez, E., Rieur, O., Fray-Witzer, M., & Pigeon, W. R. (2024). Insomnia partially mediates the relationship between anxiogenic and depressogenic traits and state anxiety and depression in college students. *Journal of American College Health*. https://doi.org/10.1080/07448481.2024.2427059

Sehgal, S. K., Nijhawan, A., Nijhawan, M., Singh, R. P., Agarwal, R., & Sardana, P. (2016). To study the sleep habits and prevalence of sleep disorders in the adolescent and adult paramedical personnel - a questionnaire-based study. *Journal Of Evolution Of Medical And Dental Sciences*, *5*(87), 6501-6507. https://doi.org/10.14260/jemds/2016/1470

Serafin, L. I., Fukowska, M., Zyskowska, D., Olechowska, J., & Czarkowska-Paczek, B. (2021). Impact of stress and coping strategies on insomnia among Polish novice nurses who are employed in their field while continuing their education: a cross-sectional study. *BMJ Open*, *11*(12), Article e049787. https://doi.org/10.1136/bmjopen-2021-049787

Shakeel, H. A., Maqsood, H., Ishaq, A., Ali, B., Hussain, H., Khan, A. R., & Shah, S. (2019). Insomnia among medical students: a cross-sectional study. *International Journal of Research in Medical Sciences*, *7*(3), 893. https://doi.org/10.18203/2320-6012.ijrms20190944

Sharma, K. K., Kaur, R., Srinivasan, M., Sarkar, S., Mani, K., Sharma, Y., & Gupta, S. (2021). Impact of COVID-19 on mental health of healthcare professionals working in COVID-19 designated clinical areas in India. *International Journal Of Community Medicine And Public Health*, *8*(3), 1406-1414. https://doi.org/10.18203/2394-6040.ijcmph20210835

Sheaves, B., Porcheret, K., Tsanas, A., Espie, C. A., Foster, R. G., Freeman, D., Harrison, P. J., Wulff, K., & Goodwin, G. M. (2016). Insomnia, nightmares, and chronotype as markers of risk for severe mental illness: Results from a student population [Physical & Somatic Disorders 3290]. *Sleep: Journal of Sleep and Sleep Disorders Research*, *39*(1), 173-181. https://doi.org/10.5665/sleep.5342

Shen, Y., Jin, X., Zhang, Y., Huang, C., Lu, J., Luo, X., & Zhang, X. Y. (2020). Insomnia in Chinese College Students

With Internet Addiction: Prevalence and Associated Clinical Correlates. *Frontiers in Psychiatry*, *11*, 596683. https://doi.org/10.3389/fpsyt.2020.596683

Shen, Y., Wang, L., Huang, C., Guo, J., De Leon, S. A., Lu, J., Luo, X., & Zhang, X. Y. (2021). Sex differences in prevalence, risk factors and clinical correlates of internet addiction among chinese college students [Behavior Disorders & Antisocial Behavior 3230]. *Journal of Affective Disorders*, *279*, 680-686. https://doi.org/10.1016/j.jad.2020.10.054

Shi, C., Wang, S., Tang, Q., Liu, X., & Li, Y. (2022). Cross-lagged relationship between anxiety, depression, and sleep disturbance among college students during and after collective isolation. *Frontiers In Public Health*, *10*, 1038862. https://doi.org/10.3389/fpubh.2022.1038862

Shi, X., Zhu, Y., Wang, S., Wang, A., Chen, X., Li, Y., & Jiang, L. (2021). The prospective associations between different types of sleep disturbance and suicidal behavior in a large sample of chinese college students. *Journal of Affective Disorders*, *279*, 380-387. https://doi.org/10.1016/j.jad.2020.10.019

Siddiqui, A. F., Al-Musa, H., Al-Amri, H., Al-Qahtani, A., Al-Shahrani, M., & Al-Qahtani, M. (2016). Sleep Patterns and Predictors of Poor Sleep Quality among Medical Students in King Khalid University, Saudi Arabia. *Malaysian Journal Of Medical Science*, *23*(6), 94-102. https://doi.org/10.21315/mjms2016.23.6.10

Sing, C. Y., & Wong, W. S. (2010). Prevalence of insomnia and its psychosocial correlates among college students in Hong Kong [Physical & Somatic Disorders 3290]. *Journal of American College Health*, *59*(3), 174-182. https://doi.org/10.1080/07448481.2010.497829

Sing, C. Y., & Wong, W. S. (2011). The effect of optimism on depression: The mediating and moderating role of insomnia [Affective Disorders 3211]. *Journal Of Health Psychology*, *16*(8), 1251-1258. https://doi.org/10.1177/1359105311407366

Siomos, K. E., Braimiotis, D., Floros, G. D., Dafoulis, V., & Angelopoulos, N. V. (2010). Insomnia symptoms among Greek adolescent students with excessive computer use. *Hippokratia*, *14*(3), 203-207.

Sivertsen, B., Hysing, M., Harvey, A. G., & Petrie, K. J. (2021). The epidemiology of insomnia and sleep duration across mental and physical health: The SHoT study. *Frontiers In Psychology*, *12*. https://doi.org/10.3389/fpsyg.2021.662572

Sivertsen, B., Vedaa, Ø., Harvey, A. G., Glozier, N., Pallesen, S., Aarø, L. E., Lønning, K. J., & Hysing, M. (2019). Sleep patterns and insomnia in young adults: A national survey of Norwegian university students. *Journal of Sleep Research*, *28*(2), e12790. https://doi.org/10.1111/jsr.12790

Snyder, M., Alldredge, C. T., Stork, S. R., & Elkins, G. R. (2023). Feasibility of a Self-Administered Hypnosis Intervention for Improving Sleep in College Students. *International Journal of Clinical and Experimental Hypnosis*, *71*(4), 297-312. https://doi.org/10.1080/00207144.2023.2249047

Solanki, S., Venkiteswaran, A., & Saravanabawan, P. (2023). Prevalence of Insomnia and Factors Influencing Its Incidence in Students of Tbilisi State Medical University: A Cross-Sectional Study. *Cureus Journal Of Medical Science*, *15*(9), Article e46084. https://doi.org/10.7759/cureus.46084

Sredniawa, A., Drwila, D., Krotos, A., Wojtas, D., Kostecka, N., & Tomasik, T. (2019). Insomnia and the level of stress among students in Krakow, Poland. *Trends In Psychiatry And Psychotherapy*, *41*(1), 60-68. https://doi.org/10.1590/2237-6089-2017-0154

Steine, I. M., Skogen, J. C., Hysing, M., Puigvert, L., Schonning, V., & Sivertsen, B. (2021). Sexual harassment and assault predict sleep disturbances and is partly mediated by nightmares: Findings from a national survey of all university students in Norway. *Journal of Sleep Research*, *30*(6). https://doi.org/10.1111/jsr.13338

Suganya, E., Arvinth, A., & Vedapriya, D. R. (2022). Sleep Hygiene Intervention and its Effectiveness in Reduction of Insomnia and Obesity among Undergraduate Medical Students. *Journal of Clinical and Diagnostic Research*, *16*(8), LC18-LC21. https://doi.org/10.7860/JCDR/2022/55304.16727

Sun, C. Q., Zhu, Z. Q., Zhang, P. J., Wang, L. K., Zhang, Q., Guo, Y. L., Guo, L. N., Li, Y., Wang, P. P., Hu, B., Liu, M. T., Duan, J. Y., Wang, Y. W., Wang, Z. Q., & Qin, Y. (2024). Exploring the interconnections of anxiety, depression, sleep problems and health-promoting lifestyles among Chinese university students: a comprehensive network approach. *Frontiers in Psychiatry*, *15*, Article 1402680. https://doi.org/10.3389/fpsyt.2024.1402680

Syafriani, N., Lailiyya, N., & Nurhayati, T. (2015). The prevalence of insomnia and the description of sleep hygiene in medical students, Universitas Padjadjaran, Indonesia. *Sleep Medicine*, *16*, S104-S104. https://doi.org/10.1016/j.sleep.2015.02.1387

Syed, W., & Al-Rawi, M. B. A. (2023). Assessment of Sleeping Disorders, Characteristics, and Sleeping Medication Use Among Pharmacy Students in Saudi Arabia: A Cross-Sectional Quantitative Study. *Medical Science Monitor*, *29*, e942147. https://doi.org/10.12659/MSM.942147

Tadros, M., Li, S., Corkish, B., Upton, E., Newby, J., & Werner-Seidler, A. (2024). Cognitive behavior therapy for insomnia in university students delivered via videoconferencing groups: A pilot study. *Behavioral Sleep Medicine*, *22*(6). https://doi.org/10.1080/15402002.2024.2374258

Tafoya, S. A., Jurado, M. M., Yépez, N. J., Fouilloux, M., & Lara, M. C. (2013). Sleep difficulties and psychological symptoms in medicine students in Mexico. *Medicina-Buenos Aires*, *73*(3), 247-251.

Takuyo, C., Yusuke, H., Toru, H., Yasuhiro, K., Shunya, I., Danya, K., Takahiro, I., & Takashi, S. (2023). Prevalence and Risk Factors of Insomnia and Sleep-aid Use in Emergency Physicians in Japan: Secondary Analysis of a Nationwide Survey. *Western Journal of Emergency Medicine: Integrating Emergency Care with Population Health*, *24*(2), 331-339. https://doi.org/10.5811/westjem.2022.12.57910

Tang, N. K. Y., McEnery, K. A. M., Chandler, L., Toro, C., Walasek, L., Friend, H., Gu, S., Singh, S. P., & Meyer, C. (2022). Pandemic and student mental health: mental health symptoms among university students and young adults after the first cycle of lockdown in the UK. *BJPsych Open*, *8*(4), e138. https://doi.org/10.1192/bjo.2022.523

Tang, Q. H., Zou, X. Y., Li, Y. F., Xu, Y. Y., Lv, Y. C., Liu, X. P., Liu, G., & Tao, Y. Q. (2024). Insomnia mediates the relation between empathy and anxiety among nursing students: a latent moderated mediation model of self-compassion. *BMC Nursing*, *23*(1), Article 570. https://doi.org/10.1186/s12912-024-02238-8

Taylor, D. J., Bramoweth, A. D., Grieser, E. A., Tatum, J. I., & Roane, B. M. (2013). Epidemiology of insomnia in college students: Relationship with mental health, quality of life, and substance use difficulties [Psychological & Physical Disorders 3200]. *Behavior Therapy*, *44*(3), 339-348. https://doi.org/10.1016/j.beth.2012.12.001

Taylor, D. J., Gardner, C. E., Bramoweth, A. D., Williams, J. M., Roane, B. M., Grieser, E. A., & Tatum, J. I. (2011). Insomnia and mental health in college students [Physical & Somatic Disorders 3290]. *Behavioral Sleep Medicine*, *9*(2), 107-116. https://doi.org/10.1080/15402002.2011.557992

Thomas, S. J. (2015). A survey of sleep disorders in college students: A study of prevalence and outcomes [Health & Mental Health Treatment & Prevention 3300]. *Dissertation Abstracts International: Section B: The Sciences and Engineering*, *76*(2-B(E)), No-Specified. (Dissertation Abstracts International)

Thomas, S. J., & Lichstein, K. L. (2014). The prevalence and impact of sleep disorders in college students. *Sleep*, *37*(SUPPL. 1), A169.

Tsai, L. L., & Li, S. P. (2004). Sleep patterns in college students - Gender and grade differences. *Journal of Psychosomatic Research*, *56*(2), 231-237. https://doi.org/10.1016/S0022-3999(03)00507-5

Tsou, M. T., & Chang, B. C. C. (2019). Association of depression and excessive daytime sleepiness among sleep-deprived college freshmen in northern Taiwan. *International Journal of Environmental Research and Public Health*, *16*(17), 3148. https://doi.org/10.3390/ijerph16173148

Turki, M., Firas, J., Mhiri, H. E., Sahnoun, F., Guermazi, A., Ellouze, S., Halouani, N., & Aloulou, J. (2023). Sleep disturbances among university students : A Tunisian study. *European Psychiatry*, *66*(Supplement 1), S1101. https://doi.org/10.1192/j.eurpsy.2023.2339

Vedaa, Ø., Erevik, E. K., Hysing, M., Hayley, A. C., & Sivertsen, B. (2019). Insomnia, sleep duration and academic performance: a national survey of Norwegian college and university students. *Sleep Medicine: X*, *1*, 100005. https://doi.org/10.1016/j.sleepx.2019.100005

Veldi, M., Aluoja, A., & Vasar, V. (2005). Sleep quality and more common sleep-related problems in medical students. *Sleep Medicine*, *6*(3), 269-275. https://doi.org/10.1016/j.sleep.2004.12.003

Virnoche, R., Gratton, M., Baber, G., & Hamilton, N. (2024). Bisexual women are more prone to nightmares and insomnia compared to straight women in a sample of college students. *Sleep*, *47*(Supplement 1), A313. https://doi.org/10.1093/sleep/zsae067.0731

Wang, D., Zhao, J., Zhai, S., Huang, S., Yang, Z., Pan, Y., Liu, X., & Fan, F. (2022). Longitudinal trajectories of insomnia symptoms among college students during the COVID-19 lockdown in China [Physical & Somatic Disorders 3290

Health Psychology & Medicine 3360]. *Journal of Psychosomatic Research*, *157*, 1-8. https://doi.org/10.1016/j.jpsychores.2022.110795

Wang, H. Q., & Sun, H. L. (2024). The mediating role of rumination in the relationship between insomnia and non-suicidal self-injury of college students. *Frontiers In Psychology*, *15*. https://doi.org/10.3389/fpsyg.2024.1504890

Wang, J., Xu, X., Zuo, L., Wang, H., & Yang, G. (2024). Mobile phone addiction and insomnia among college students in China during the COVID-19 pandemic: a moderated mediation model. *Frontiers In Public Health*, *12*, 1338526. https://doi.org/10.3389/fpubh.2024.1338526

Wang, L., Qin, P., Zhao, Y. S., Duan, S. Y., Zhang, Q., Liu, Y., Hu, Y. L., & Sun, J. (2016). Prevalence and risk factors of poor sleep quality among Inner Mongolia Medical University students: A cross-sectional survey. *Psychiatry Research*, *244*, 243-248. https://doi.org/10.1016/j.psychres.2016.04.011

Wang, S., Julich, S. T., & Lei, X. (2024). Latent profile of the Insomnia Severity Index: A longitudinal study [Clinical Psychological Testing 2224 Physical & Somatic Disorders 3290]. *Sleep Medicine*, *115*, 202-209. https://doi.org/10.1016/j.sleep.2024.02.027

Williams, A. B., Dzierzewski, J. M., Griffin, S. C., Lind, M. J., Dick, D., & Rybarczyk, B. D. (2020). Insomnia disorder and behaviorally induced insufficient sleep syndrome: Prevalence and relationship to depression in college students [Psychological & Physical Disorders 3200]. *Behavioral Sleep Medicine*, *18*(2), 275-286. https://doi.org/10.1080/15402002.2019.1578772

Wu, R., Wang, C.-Y., Wang, F., Wang, Y.-J., Zhu, H., Wang, G.-H., & Jiang, C.-L. (2022). Association between Sleep and Suicidal Ideation in Chinese Undergraduate Students. *International Journal of Environmental Research and Public Health*, *19*(23). https://doi.org/10.3390/ijerph192315433

Xiao, S., Liu, S., Zhang, P., Yu, J., Huaihong, A., Wu, H., Zhang, F., Xiao, Y., Ma, N., Ma, X., Li, J., Wang, X., Shao, X., Liu, W., Zhang, X., Wu, W., Wang, L., Wu, R., He, Y.,…Zhang, B. (2021). The Association Between Depressive Symptoms and Insomnia in College Students in Qinghai Province: The Mediating Effect of Rumination. *Frontiers in Psychiatry*, *12*, 751411. https://doi.org/10.3389/fpsyt.2021.751411

Xu, H., Tao, J., Yang, J., Su, Y., Xu, C., Hu, M., Lum, G. G. A., Hu, D., & Lu, L. (2023). Mobile phone use addiction, insomnia, and depressive symptoms in adolescents from ethnic minority areas in China: A latent variable mediation model. *Journal of Affective Disorders*, *320*, 381-389. https://doi.org/10.1016/j.jad.2022.09.156

Xu, Y., Su, S., Jiang, Z., Guo, S., Lu, Q., Liu, L., Zhao, Y., Wu, P., Que, J., Shi, L., Deng, J., Meng, S., Yan, W., Sun, Y., Yuan, K., Lin, X., Sun, S., Ravindran, A. V., Chen, S.,…Lu, L. (2021). Prevalence and Risk Factors of Mental Health Symptoms and Suicidal Behavior Among University Students in Wuhan, China During the COVID-19 Pandemic. *Frontiers in Psychiatry*, *12*, 695017. https://doi.org/10.3389/fpsyt.2021.695017

Yavuz, F., Kabaagil, B., Ismailogullari, S., Zararsiz, G., & Per, H. (2019). Investigation of the Prevalence of Sleep Disorders in Medical Students and Examination of Its Change by Classes, Gender and Body Mass Index. *Journal of Turkish Sleep Medicine-Turk Uyku Tibbi Dergisi*, *6*(3), 88-92. https://doi.org/10.4274/jtsm.galenos.2019.46036

Yilmaz, Y., & Kugu, N. (2022). The prevalence of insomnia in university students and its relationship with quality of life: a university sample.

Younes, F., Halawi, G., Jabbour, H., El Osta, N., Karam, L., Hajj, A., & Khabbaz, L. R. (2016). Internet addiction and relationships with insomnia, anxiety, depression, stress and self-esteem in university students: A cross-sectional designed study [Behavior Disorders & Antisocial Behavior 3230]. *PLoS One*, *11*(9). https://doi.org/10.1371/journal.pone.0161126

Yu, C., Li, X., Qi, G., Yang, L., Fu, W., Yao, Q., Wei, L., Zhou, D., Zhang, X., & Zheng, H. (2021). Prevalence, Risk Factors, and Clinical Correlates of Insomnia in China College Student During the COVID-19. *Frontiers in Psychiatry*, *12*, 694051. https://doi.org/10.3389/fpsyt.2021.694051

Yu, J., Liu, K., & Liu, S. (2020). Relationship between coping style and insomnia in college students: the mediating effect of rumination. *Nan fang yi ke da xue xue bao = Journal of Southern Medical*

University, *40*(1). https://doi.org/10.12122/j.issn.1673-4254.2020.01.22

Yuan, J., Bai, J., Wang, Y., Wu, H., Mu, Y., Zhao, C., & Zhu, B. (2024). Sleep behaviors predicted sleep disturbances among Chinese health science students: a cross-sectional study. *Sleep and Breathing*, *28*(1), 449 - 457. https://doi.org/10.1007/s11325-023-02888-z

Yun-Yi Yang, & Jun, S. (2018). Prevalence and Associated Factors of Insomnia in College Students with Irritable Bowel Syndrome. *Korean Journal of Adult Nursing*, *30*(3), 235-244. https://doi.org/10.7475/kjan.2018.30.3.235

Yurasek, A. M., Miller, M. B., Pritschmann, R. K., Curtis, A. F., & McCrae, C. S. (2020). Negative mood as a mediator of the association between insomnia severity and marijuana problems in college students. *Journal of Sleep Research*, *29*(4), Article e12985. https://doi.org/10.1111/jsr.12985

Zafar, N., Kausar, R., & Pallesen, S. (2018). Internet addiction, insomnia and mental health problems in university students in Pakistan [Psychological & Physical Disorders 3200]. *Pakistan Journal of Social and Clinical Psychology*, *16*(2), 10-16.

Zainab, S., Soomro, R. A., Khoso, A., Qazi, N. A., & Siddiqui, S. (2020). Frequency and predictors of sleep disorders in undergraduate medical students. *Journal of the Liaquat University of Medical and Health Sciences*, *19*(2). https://doi.org/10.22442/jlumhs.201920672

Zhai, K., Gao, X., & Wang, G. (2018). The Role of Sleep Quality in the Psychological Well-Being of Final Year UndergraduateStudents in China. *International Journal of Environmental Research and Public Health*, *15*(12). https://doi.org/10.3390/ijerph15122881

Zhang, D., Qu, Y., Zhai, S., Li, T., Xie, Y., Tao, S., Zou, L., Tao, F., & Wu, X. (2023). Association between healthy sleep patterns and depressive trajectories among college students: a prospective cohort study. *BMC Psychiatry*, *23*(1), 182. https://doi.org/10.1186/s12888-023-04596-0

Zhang, W., Yu, M., Xu, Y., Li, X., Zuo, H., Huang, Z., & Gao, X. (2023). Self-reported sleep status and influencing factors: a web-based national cross-sectional survey in China. *Annals of Medicine*, *55*(2), 2287706. https://doi.org/10.1080/07853890.2023.2287706

Zhang, X., Wang, F., Zou, L. N., & Lee, S. Y. (2024). Depressive symptoms, sleep-wake features, and insomnia among female students: The role of rumination. *Journal of Health Psychology*. https://doi.org/10.1177/13591053241258252

Zhang, Y., Wang, D., Zhao, J., Chen, X.-Y., Chen, H., Ma, Z., Yu, Q., Wang, T., Chen, Z., Jiang, J., Zhu, Z., Huang, S., Fan, F., & Liu, X. (2021). Insomnia and other sleep-related problems during the remission period of the COVID-19 pandemic: A large-scale survey among college students in China [Physical & Somatic Disorders 3290]. *Psychiatry Research*, *304*. https://doi.org/10.1016/j.psychres.2021.114153

Zhao, G., Xie, F., Li, S., Ding, Y., Li, X., & Liu, H. (2022). The relationship between perceived social support with anxiety, depression, and insomnia among Chinese college students during the COVID-19 pandemic: The mediating role of self-control. *Frontiers in Psychiatry*, *13*, 994376. https://doi.org/10.3389/fpsyt.2022.994376

Zheng, X., Guo, Y., Ma, W., Yang, H., Luo, L., Wen, L., Zhou, X., Li, Q., Bi, J., Wang, P., & Wang, H. (2021). A Longitudinal Study on the Mental Health of College Students in Jinan during the Peak Stage of the

COVID-19 Epidemic and the Society Reopening. *Biomedicine Hub*, *6*(3). https://doi.org/10.1159/000519586

Zhou, J., Xing, X., Xue, P., & Tang, X. (2019). Association between sleep and menstrual problems in chinese female university students. *Sleep*, *42*(Supplement 1), A270. https://doi.org/10.1093/sleep/zsz067.672

Zou, H., Huang, J., Zhang, W., Wu, J., Wu, W., & Huo, L. (2023). The effect of cyberbullying victimization and traditional bullying victimization on suicidal ideation among Chinese female college students: The role of rumination and insomnia. *Journal of Affective Disorders*, *340*, 862-870. https://doi.org/10.1016/j.jad.2023.08.099

Zvolensky, M. J., Kauffman, B. Y., Bogiaizian, D., Viana, A. G., Bakhshaie, J., & Peraza, N. (2021). Worry among Latinx college students: relations to anxious arousal, social anxiety, general depression, and insomnia. *Journal of American College Health*, *69*(5), 529-536. https://doi.org/10.1080/07448481.2019.1686004
